# Bellymount enables longitudinal, intravital imaging of abdominal organs and the gut microbiota in adult *Drosophila*

**DOI:** 10.1371/journal.pbio.3000567

**Published:** 2020-01-27

**Authors:** Leslie Ann Jaramillo Koyama, Andrés Aranda-Díaz, Yu-Han Su, Shruthi Balachandra, Judy Lisette Martin, William B. Ludington, Kerwyn Casey Huang, Lucy Erin O’Brien

**Affiliations:** 1 Department of Molecular and Cellular Physiology, Stanford University School of Medicine, Stanford, California, United States of America; 2 Department of Developmental Biology, Stanford University School of Medicine, Stanford, California, United States of America; 3 Department of Bioengineering, Stanford University, Stanford, California, United States of America; 4 Department of Embryology, Carnegie Institution of Washington, Baltimore, Maryland, United States of America; 5 Department of Microbiology and Immunology, Stanford University School of Medicine, Stanford, California, United States of America; 6 Chan Zuckerberg Biohub, San Francisco, California, United States of America; University of Utah, UNITED STATES

## Abstract

Cell- and tissue-level processes often occur across days or weeks, but few imaging methods can capture such long timescales. Here, we describe Bellymount, a simple, noninvasive method for longitudinal imaging of the *Drosophila* abdomen at subcellular resolution. Bellymounted animals remain live and intact, so the same individual can be imaged serially to yield vivid time series of multiday processes. This feature opens the door to longitudinal studies of *Drosophila* internal organs in their native context. Exploiting Bellymount’s capabilities, we track intestinal stem cell lineages and gut microbial colonization in single animals, revealing spatiotemporal dynamics undetectable by previously available methods.

## Abstract 2

An innovative imaging platform overcomes the challenge presented by the opacity of the adult Drosophila abdomen, enabling intravital imaging of live, intact flies at whole-organ and subcellular scales over multiple days. Longitudinal tracking of stem cell lineages and gut microbial colonization reveals new dynamics of these processes in vivo.

## Introduction

A major thrust of modern biology is leveraging advances in live microscopy to reveal how cellular and physiological processes unfold inside living organisms. For adult metazoans, this goal requires overcoming two imaging challenges: the opacity of many mature animals to light and the prolonged timescales of adult-associated processes such as aging.

The adult vinegar fly, *Drosophila melanogaster*, has yielded foundational insights into metazoan physiology [1–4]. This invertebrate is also a powerful tool for probing human pathologies, with approximately 65% of human disease-causing genes having functional homologs in the fly [5]. However, current methods for imaging *Drosophila* abdominal organs are limited in optical resolution, imaging duration, or both. Some newer approaches preserve animal viability but cannot visualize individual cells [6–8]. Other recent advances enable high-resolution imaging but require opening the abdominal cuticle, which leads to eventual organismal death [9–12].

Here, we present Bellymount, a method for high-resolution imaging of the intact *Drosophila* abdomen in live adults. Bellymount captures volumetric images of native abdominal organs at spatial scales ranging from subcellular (<1 μm) to multiorgan (>100 μm). It preserves organismal viability, thereby enabling slow processes to be studied longitudinally within single animals. It is inexpensive to construct, simple to apply, and compatible with diverse brightfield and fluorescence microscopes, including both inverted and upright setups. Finally, Bellymount is easily combined with *Drosophila*’s sophisticated tools for spatiotemporal genetic manipulation, fluorescence labeling, and live reporter assays. Exploiting all these features, we use Bellymount to perform longitudinal tracking of two multiday processes in the *Drosophila* gut: generation of intestinal stem cell lineages and colonization by commensal bacteria. The resulting time series provide the first direct views of spatial and temporal heterogeneities that underlie both events. These findings demonstrate the capability of Bellymount to uncover new physiological dynamics of cells, tissues, and organs in vivo.

## Results

**Fig 1:**
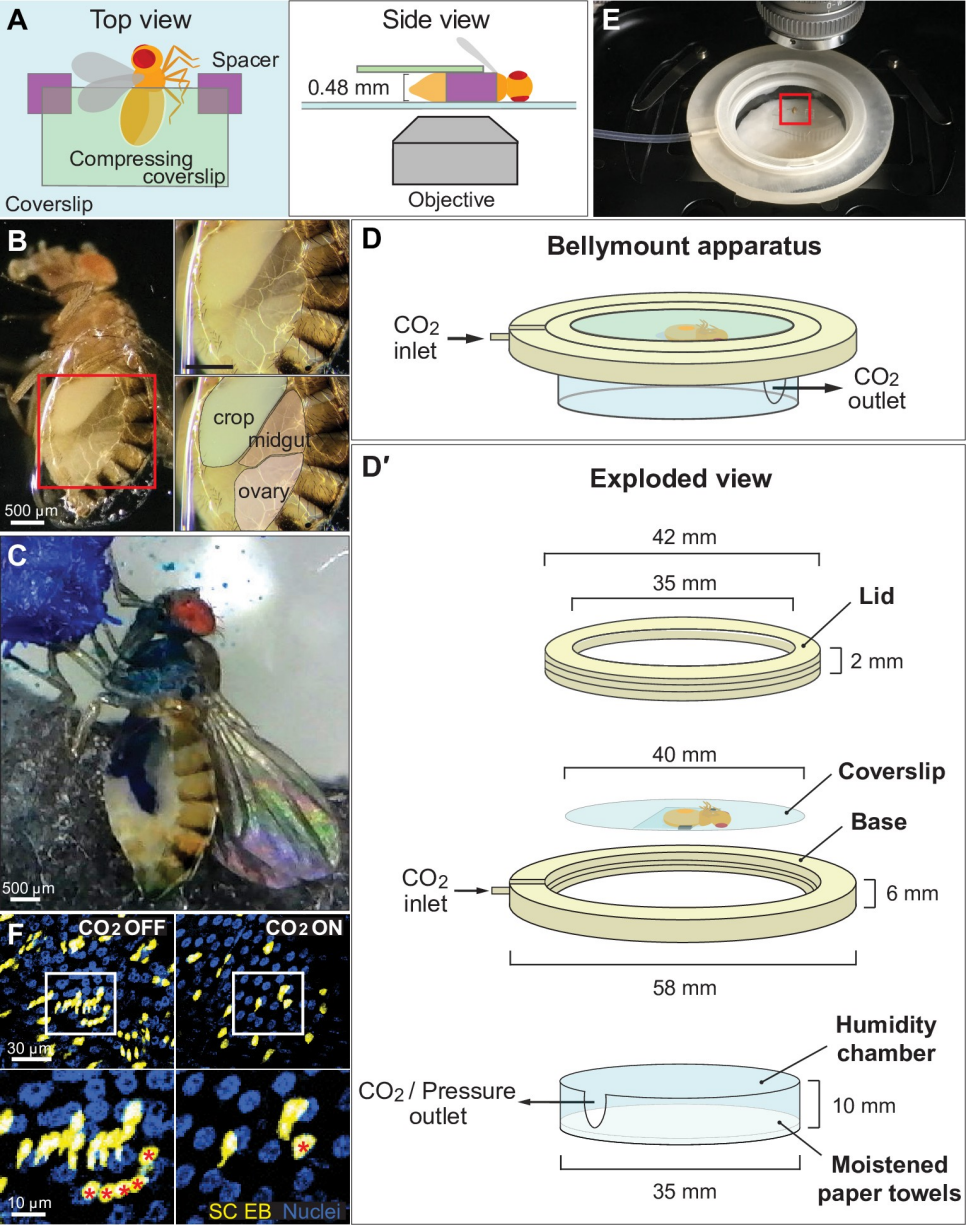
The Bellymount platform enables intravital imaging of the adult *Drosophila* abdomen. (A) Cartoons of Bellymounted animal. Top and side views are shown. The cuticle of the ventrolateral abdomen is glued to the bottom coverslip. To maximize contact between the cuticle and the glue, a second, smaller coverslip gently compresses the abdomen. (B) Underside view of a Bellymounted adult female. Gluing caused the ventral cuticle to become light transparent. The edge of the glue patch appeared as a refractive line around the abdomen. Right panels are close-ups of boxed region in left panel. (C) Bellymounted animals ingest nutrients, undergo GI transit, and defecate. As a mounted animal ingested blue-colored sugar water, its midgut gradually became colored blue. Image shows a single time point from S3 Movie. (D) Cartoon of Bellymount apparatus for delivery of CO_2_ anesthesia during confocal imaging. Isometric drawing shows assembled (D) and exploded (D′) configurations. The coverslip with the glued animal sits inside the base of the apparatus. CO_2_ flows through the indicated ports. CAD files for 3D printing of the lid and base are provided as S1 and S2 Files. See also S2 Fig. (E) Bellymount apparatus positioned for imaging on an upright microscope. Red box shows the location of the glued animal. (F) CO_2_ anesthesia minimizes tissue movements during acquisition of confocal *z*-stacks. Without CO_2_ (left), tissue movements during *z*-stack acquisition caused individual cells to be represented multiple times. With CO_2_ (right), tissue movements were inhibited, and *z*-stack images were accurate. Bottom panels are close-ups of boxed regions in top panels. Red asterisks label the identical cell in images taken without and with CO_2_. Blue, nuclei; yellow, midgut stem cells and enteroblasts. Images are projections of confocal stacks. Genotype: *esg>LifeActGFP; ubi-his2av*::*mRFP*. See S4 Movie. CAD, computer-aided design; EB, enteroblast; *esg*, *escargot-*Gal4; *GFP*, green fluorescent protein; GI, gastrointestinal; *his2av*, histone variant His2av; *mRFP*, monomeric red fluorescent protein; *RFP*, red fluorescent protein; SC, stem cell.

The exterior cuticle of the adult fly, which is generally opaque, presents an obstacle for light-based imaging of internal organs. Serendipitously, we noticed that the ventral abdominal cuticle becomes transparent when affixed to a glass coverslip by the polyvinyl acetate adhesive, Elmer’s Clear School Glue (Fig 1A and 1B and S1 Movie). We named this procedure “Bellymount.” The transparency of the glued cuticle enabled facile observation of organs such as the midgut, crop, and female ovaries in flies that were live and intact (Fig 1B). Bellymounted animals were readily removed from the coverslip, even hours after the glue had dried (S2 Movie). Furthermore, these animals were generally viable; in a survival assay, 92% of animals were alive 24 h after being glued and released (S1 Fig).

The abdomen is the body’s central location for digestive physiology and function. To explore the utility of Bellymount for gastrointestinal (GI) studies, we used an inexpensive, universal serial bus (USB)-pluggable microscope to record food ingestion and transit. Bellymounted animals were provided 5% sucrose water that was colored with Brilliant Blue FCF. Over 45 min, the ingested blue liquid filled successive compartments of the GI tract, with rapid peristaltic contractions accompanying nutrient transit (Fig 1C and S3 Movie). The vivid spatiotemporal resolution of these digestive events demonstrates the potential of Bellymount to investigate GI function in real time.

We next assessed whether cells in abdominal organs could be resolved individually. Using fluorescence confocal microscopy, cells in the midgut were observed readily, but digestive peristalsis and global body movements caused single cells to be recorded multiple times (Fig 1F, left panel and S4 Movie). To overcome this obstacle, we designed a 3D-printed apparatus for administration of carbon dioxide (CO_2_) anesthesia to Bellymounted flies. The Bellymount apparatus comprises 3 parts: a base that holds the coverslip with the mounted animal, a screw-on lid, and a humidity chamber to ensure that the animal does not desiccate during imaging (Fig 1D and 1E, S2 Fig, and S1 and S2 Files). CO_2_, delivered through an inlet in the base, inhibited both midgut peristalsis and overall body movement. This effect enabled acquisition of crisp confocal *z-*stacks at single-cell resolution (Fig 1F, right panel, and S4 Movie).

**Fig 2:**
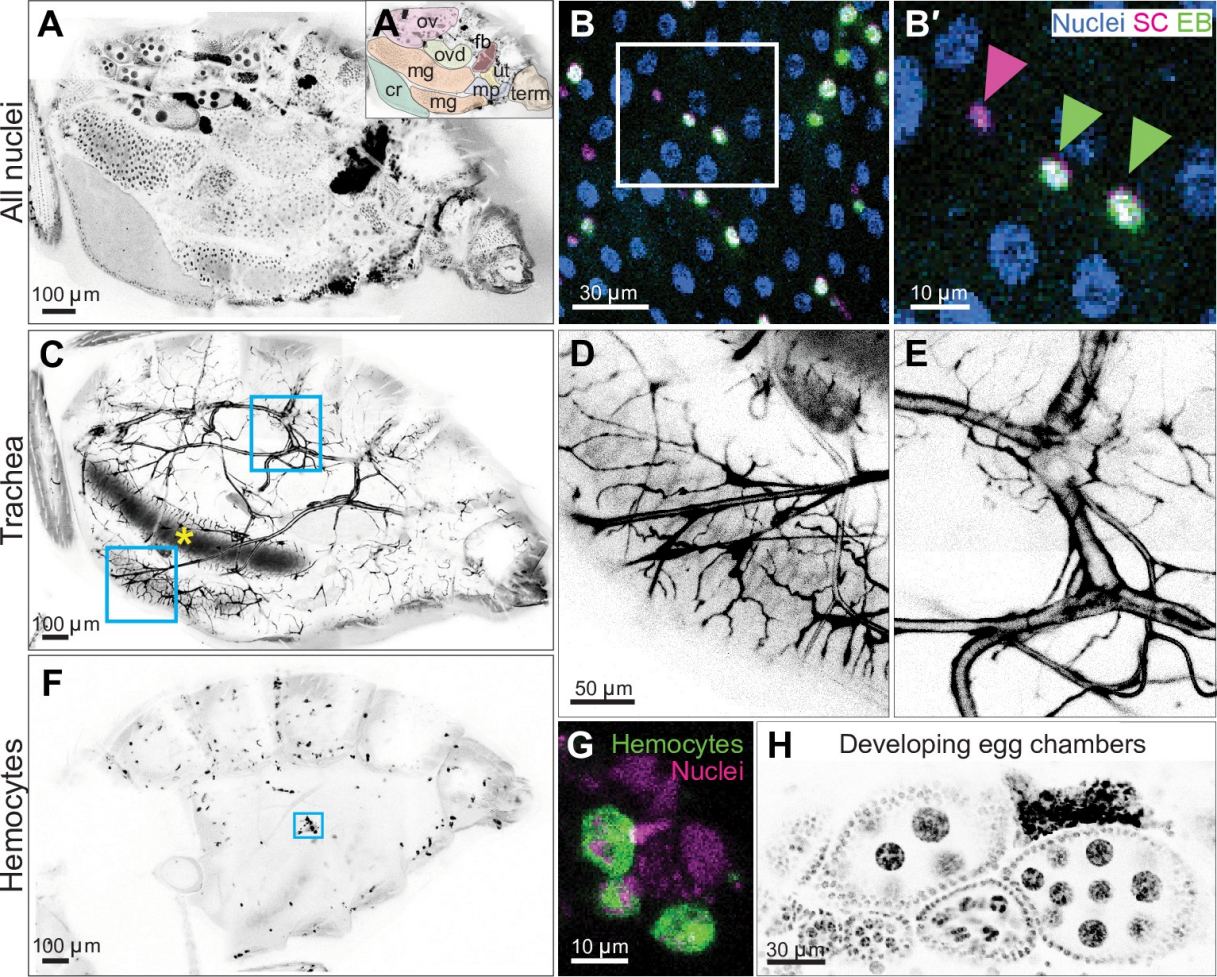
Whole-abdomen, micron-resolution imaging of organs, tissues, and cells in live, intact animals. (A) Native arrangement of abdominal organs. Visible organs include the crop (cr), midgut (mg), ovary (ov), oviduct (ovd), fat body (fb), uterus (ut), Malphigian tubules (mp), and terminalia (term). Inverted grayscale, nuclei. Genotype: *esg>his2b*::*CFP*, *GBE-Su(H)-GFP*::*nls; ubi-his2av*::*mRFP* (only RFP is shown). See S5 Movie. (B) Nuclear morphologies of individual midgut cells. Immature diploid cells (SCs [magenta nuclei] and terminal EBs [green nuclei]) were dispersed among mature, polyploid enterocytes (blue nuclei). Right panel is a close-up of boxed region in left panel. Arrowheads indicate nuclei of a stem cell (magenta) and two enteroblasts (green). Genotype: *esg>his2b*::*CFP*, *GBE-Su(H)-GFP*::*nls; ubi-his2av*::*mRFP*. (C) The abdominal tracheal network. Whole-abdomen imaging showed connectivity of tracheal branches from cuticular spiracles to internal organs. Ingested food in the midgut lumen exhibited autofluorescence (yellow asterisk). Boxed areas are shown as close-ups in D and E. (D) An extensive network of secondary and tertiary trachea wrap around the midgut tube. (E) Primary trachea exhibit branching near their origin at the cuticular spiracle. Genotype in C–E: *breathless>GFP*; ubi-his2av::mRFP (only GFP is shown). (F) Whole-abdomen distribution of hemocytes. Individual hemocytes tended to localize to pigmented regions of abdominal tergites. (G) Morphology of individual hemocytes. Image is a close-up of boxed region in F. Scale bar, 30 μm. Genotype in F–G: *hml>GFP*; *ubi-his2av*::*mRFP* (only the GFP channel is shown in F). (H) Developing egg chambers. Each chamber included the immature oocyte and polyploid nurse cells that are surrounded by diploid follicle cells. Four chambers, arranged by developmental stage, are aligned within one ovariole at the bottom. One chamber from a different ovariole is at the top. Nuclei, inverted grayscale. Genotype: *ubi-his2av*::*mRFP*. See S6 Movie. All images are projections of confocal stacks. *CFP*, cyan fluorescent protein; EB, enteroblast; *esg*, *escargot-*Gal4; *GBE*, Grainyhead binding element; *GFP*, green fluorescent protein; *hml*, hemolectin; *his2av*, histone variant His2av; *mRFP*, monomeric red fluorescent protein; *nls*, nuclear localization sequence; *RFP*, red fluorescent protein; SC, stem cell; *Su(H)*, Suppressor of Hairless.

By gently compressing the animal with a coverslip during anesthesia (Fig 1A and S1 Movie), we were able to view the entire abdomen at single-cell resolution. Using either confocal or two-photon microscopy, we acquired volumetric tile scans of nearly all female abdominal organs: midgut, crop, rectum, ovary, oviduct, uterus, fat body, and Malpighian tubule (Fig 2A and S5 Movie), as well as the tracheal network (Fig 2C, 2D and 2E) and circulating hemocytes (Fig 2F and 2G). Whole-abdomen scans of fed versus starved female abdomens revealed the extent to which abdominal organs are remodeled following nutrient ingestion and demonstrate Bellymount’s ability to document these changes in live animals (S8 Fig).

In the midgut, stem cells and various stages of terminal progeny were easily distinguished when labeled with fate-specific fluorescent markers (Fig 2B). In the ovary, egg chambers at various developmental stages were apparent; within these chambers, nascent oocytes and their supporting cells were readily identifiable (Fig 2H and S6 Movie). Micron-level resolution of subcellular structures such as actin filaments and puncta was easily obtained (S3 Fig). Thus, Bellymount enables—for the first time, to our knowledge, in adult *Drosophila*—observation of native abdominal organs at subcellular resolution in animals that are live, intact, and viable.

The viability of flies after Bellymount raised the possibility of performing Bellymount imaging on the same individuals across multiple days. To investigate this possibility, we considered two types of cellular events: divisions of midgut intestinal stem cells and microbial colonization of the GI tract. Both processes are conserved in vertebrates and are a focus of intense research interest. However, the real-time dynamics of these fundamental processes remain virtually unexplored because of lack of methods for multiday tracking in single animals.

The *Drosophila* midgut is physiologically equivalent to the vertebrate stomach and small intestine. As in vertebrate intestines, stem cells in the fly midgut continuously divide to replenish terminally differentiated epithelial cells that form the intestinal barrier [13]. Although current live-imaging methods capture single divisions [12], their ≤16-h timescales are insufficient to track multiple divisions of the same stem cell or to monitor differentiation of new progeny.

To track stem cell divisions, we used green fluorescent protein (GFP)-marked stem cell clones, which are regarded as the gold standard for tracing stem cell lineages [14]. The MARCM (Mosaic Analysis with a Repressible Cell Marker) system [15] was used to switch on constitutive expression of GFP in a small number of single stem cells. A progeny cell arising from these labeled stem cells inherited GFP expression from their mother stem cells. Over time, these progenies manifested as a cluster of labeled cells, termed a clone. The cellular composition of the clone represents the stem cell’s “lineage.”

Stem cell clones have traditionally been analyzed in fixed tissues. This approach provides a static snapshot of stem cell lineages. However, it cannot provide a temporal record of when progenies were born, how quickly they differentiated, or whether any progeny died. Such time-resolved information is crucial for a deep understanding of adult tissue homeostasis.

**Fig 3:**
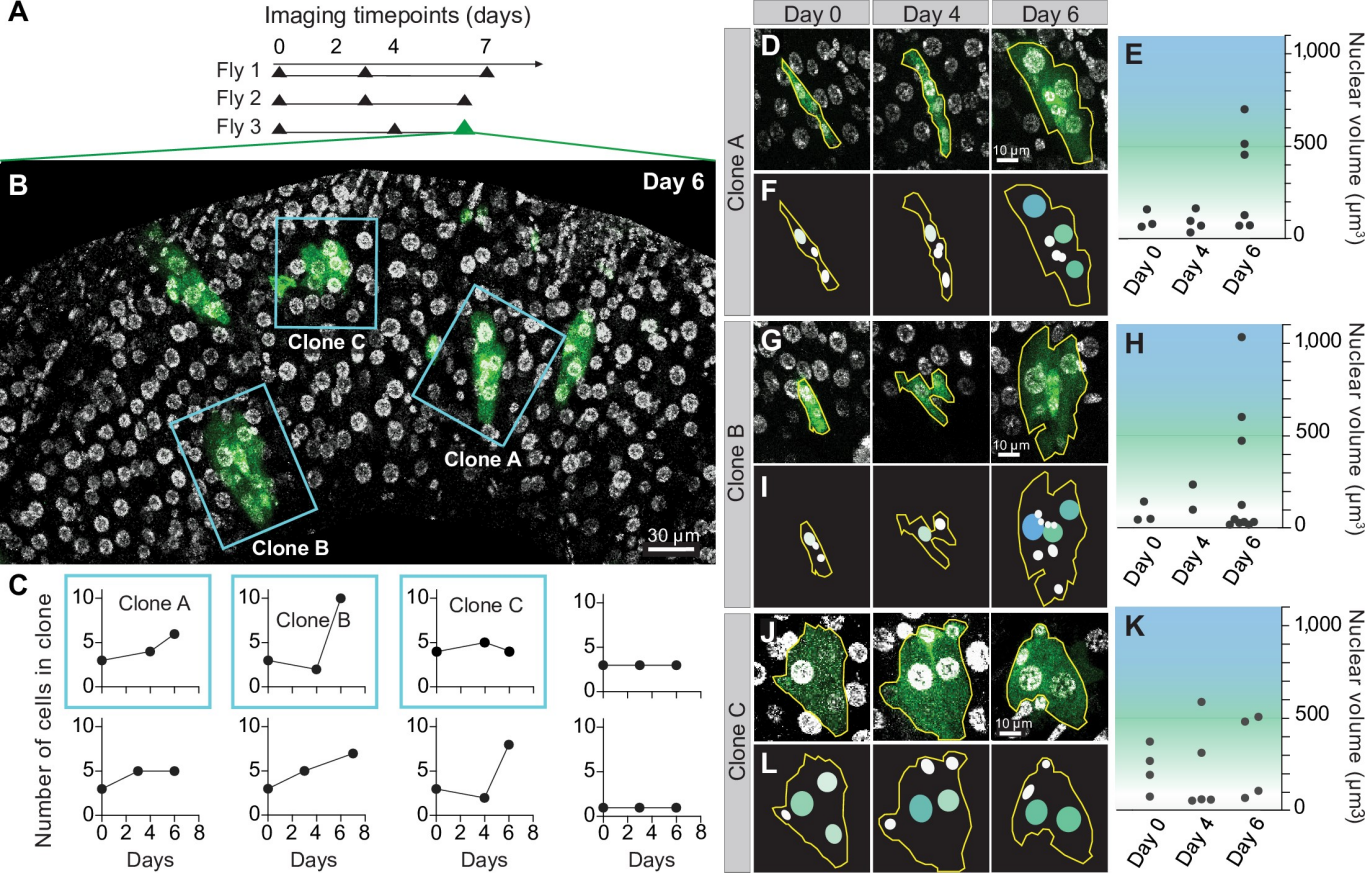
Serial imaging reveals longitudinal dynamics of midgut stem cell lineages. (A) Time points for serial imaging of GFP-marked midgut stem cell clones. Clones arose spontaneously during adult days 0–4. The first imaging time point, which was performed at adult day 4, is designated as day 0 in the experiment schematic. (B) Clones form organ-level patterns that enable their reidentification across imaging sessions. Blue boxes outline 3 midgut clones in Fly 3 that were tracked for the duration of the experiment. Green, GFP-labeled clones; grayscale, nuclei. See S4 Fig. (C) Longitudinal dynamics of clone cell addition and loss. Cells were counted at each imaging time point for 8 clones from the 3 animals in A. Between time points, numbers of cells per clone either increased, decreased, or remained constant. The data underlying this figure are included in S1 Data. (D,G,J) Time-series images of individual clones. Clones A, B, and C from Fly 3 are shown. See S4 Fig and S7 Movie. (E,H,K) Some clone cells differentiate into enterocytes during the imaging time course. Enterocyte differentiation was characterized by increased nuclear volume (green-to-blue shading; see S5 Fig). Over time, cells in clones became more likely to exhibit nuclear volumes typical of enterocytes (>300 μm^3^). The data underlying this figure are included in S1 Data. (F,I,L) Cartoons of clones. Nuclei in cartoons are color-coded to represent the measured nuclear volumes of individual clone cells. Volumes correspond to the white-green-blue color scale in E, H, and K. Genotype for all panels: *UAS-CD8-GFP*, *hs-flp12; tubGal4; FRT82*, *tubGal80/FRT82*. All images are projections of confocal stacks. *CD8*, membrane protein CD8; *FRT*, Flippase Recognition Target; *GFP*, green fluorescent protein; *hs-flp*, heat-shock flippase; *tub*, αTubulin84b promoter; *UAS*, Upstream Activation Sequence.

We asked whether the longitudinal dynamics of stem cell lineages could be tracked by serial Bellymount imaging. In MARCM animals, spontaneous recombination during the first 4 days of adult life generated sporadic GFP labeling of midgut stem cells. These founder cells subsequently developed into GFP-marked, multicellular clones (Fig 3B and S4 Fig).

To test whether individual clones could be reidentified over time, we serially imaged midguts from the same animals over multiple days (Fig 3A). In these midguts, multiple clones were reidentifiable because they exhibited unique spatial patterns or distinctive shapes (S4 Fig). Other clones could not be tracked because they lacked distinguishing morphological characteristics or disappeared from view because of slight displacements or rolling of the midgut tube.

We examined the multiday dynamics of 8 stem cell lineages. First, we determined how lineages grew or shrank by counting the number of clone cells per time point (Fig 3C). The 8 clones exhibited 3 types of trajectories: 2 clones kept the same number of cells, 3 clones added additional cells, and 3 clones both added and lost cells. Notably, cell loss is an event that is undetectable in fixed tissues. Between time points, rates of cell addition exhibited a 16-fold range of 0.25–4 cells per day. While absolute numbers of cells in our tracked clones resembled those in fixed tissue studies [16–18], our longitudinal analysis revealed that clones with similar numbers of cells can arise through trajectories that are extremely different.

Next, we considered the rates at which cells differentiated into enterocytes. In the fly midgut, a new stem cell daughter differentiates into a terminal enterocyte via an intermediate state called an enteroblast [18]. This process is characterized by increasing ploidy; stem cells and new daughters are 2N, enteroblasts are 2–8N, and enterocytes are 8–64N [19]. Hence, nuclear volume provides an indicator of how far enteroblasts and immature enterocytes have progressed toward terminal differentiation (S5 Fig).

Using nuclear volumes, we assessed differentiation rates for cells in 3 clones from one midgut (Clones A, B, and C in Fly 3; shown in organ-level view in Fig 3B and S4 Fig and in zoomed view in Fig 3D, 3G and 3J, and S7 Movie). These measurements indicated that some stem cell progeny progressed to enteroblast or enterocyte states, whereas others did not (Fig 3E, 3F, 3H, 3I, 3K and 3L). In certain cases, the data also provided more nuanced information. For instance, enterocytes were absent in Clones A and B between days 0–4 but appeared by day 6, suggesting that enterocyte differentiation can proceed rapidly once initiated. As another example, the disappearance of an enterocyte in Clone C between days 0 and 4 implies that either cell loss or dedifferentiation occurred.

Altogether, these analyses provide the first direct views of how single stem cell lineages develop. They demonstrate that the dynamics of cell addition and differentiation are extremely heterogeneous—not only between different stem cell lineages, as has been proposed previously [17,20], but also within the same lineage at different times, an intriguing feature that can be discerned only from longitudinal data. The ability of Bellymount to track individual lineages for prolonged times will facilitate future mechanistic studies of these dynamics.

We applied serial Bellymount imaging to examine a second process, colonization of the GI tract by commensal bacteria. While the human gut microbiota comprises hundreds of bacterial species, the *Drosophila* gut microbiota typically comprises only 5 [21]. This relative simplicity, together with *Drosophila*’s genetic tractability, has enabled the fly to become a powerful model for mechanistic study of host–microbiota interactions.

The biogeography, or dynamic spatial distribution, of the gut microbiota along the GI tract is known to impact host–microbiota interactions and digestive physiology [22,23]. However, the biogeography of the *Drosophila* gut is largely unknown because of the lack of methods for monitoring gut bacteria throughout the GI tracts of living animals.

**Fig 4:**
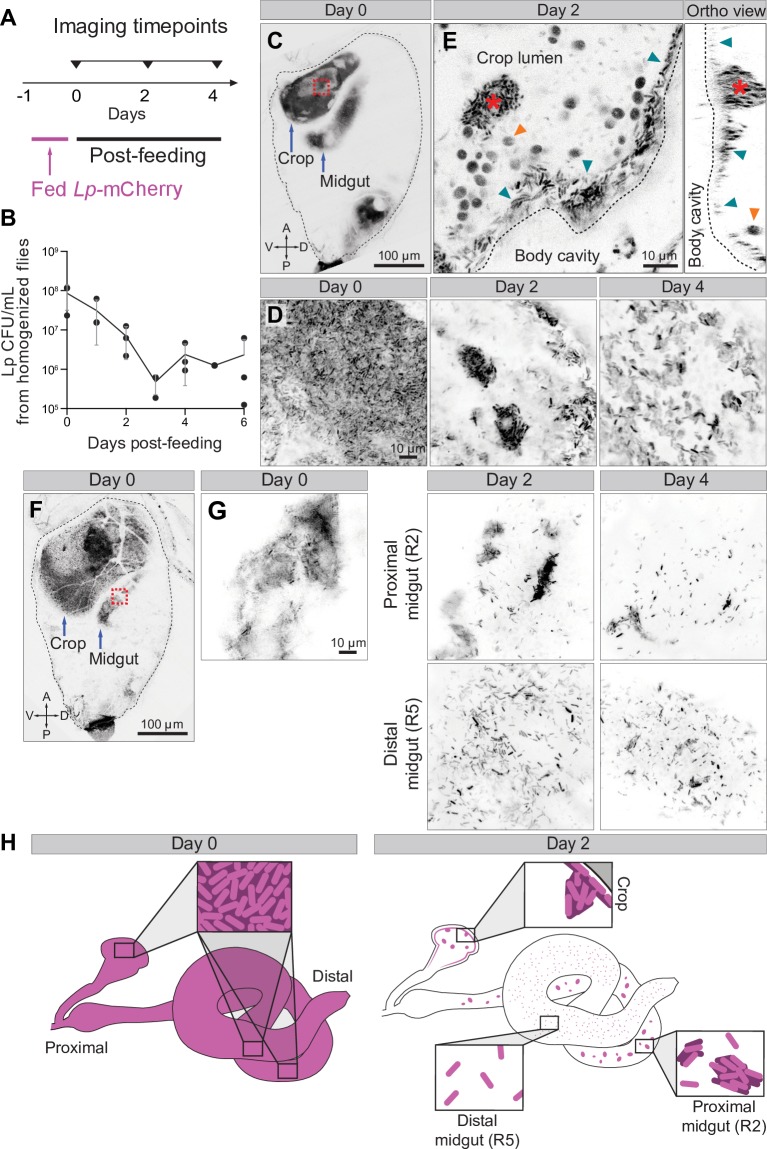
Serial imaging reveals regional dynamics of gut bacterial colonization. (A) Time points for serial imaging of gut colonization by *L*. *plantarum*. Animals were administered a 1-day pulse of *L*. *plantarum*-mCherry. *L*. *plantarum* was removed, and individual animals were imaged as indicated. The first imaging session is designated day 0. (B) Whole-animal CFUs following a 1-day pulse of *L*. *plantarum*-mCherry. Single flies were homogenized at the indicated times, and *L*. *plantarum* CFUs were measured. Each point represents CFUs from one animal. CFUs plateaued at 3 days, indicating stable colonization. The data underlying this figure are included in S2 Data. (C–E) Longitudinal imaging of *L*. *plantarum* in the crop of a single animal. (C) Whole abdomen on day 0. *L*. *plantarum*-mCherry (inverted grayscale) filled the crop and midgut. The boxed region is shown as a close-up in D. (D) Time series of *L*. *plantarum* in the crop of the animal in C. The distribution of *L*. *plantarum* was initially dense and became sparser over time. (E) Detailed images of crop from day 2. Sagittal (left) and orthogonal (right) sections are shown. *L*. *plantarum* accumulated along the crop’s inner wall (blue arrows) and formed prominent clumps (red asterisk). Ingested yeast cells (orange arrows) were also visible due to autofluorescence. See S8 Movie. (F–G) Longitudinal imaging of *L*. *plantarum* in the midgut of a single animal. (F) Whole abdomen on day 0. Inverted grayscale, *L*. *plantarum*-mCherry. Boxed region is shown as a close-up in G. (G) Time series of *L*. *plantarum* in midgut of animal in F. Only one midgut loop was visible on day 0; 2 loops (proximal/R2 and distal/R5; see S7 Fig) were visible on days 2 and 4. Over time, *L*. *plantarum* became clumped in the lumen of the proximal/R2 loop and individually dispersed in the lumen of the distal/R5 loop. See S6 Fig and S9 and S10 Movies. (H) Heterogenous dynamics of *L*. *plantarum* colonization and dispersal in distinct gut regions. Crop, proximal/R2 midgut, and distal/R5 midgut each exhibited distinct spatial patterns of bacterial localization over time. Genotype for all panels: *ubi-his2avD*::*YFP*. Images in C–F are single optical sections. Images in G are maximum projections of confocal stacks. CFU, colony-forming unit; *his2av*, histone variant His2av; *Lp*, *L*. *plantarum*; YFP, yellow fluorescent protein.

We examined whether serial Bellymount imaging could provide a direct view of gut microbial colonization. The prevalent and abundant *Drosophila* gut commensal *Lactobacillus plantarum* was tagged with mCherry [24] and fed to conventionally reared flies for 1 day. The next day, animals were removed from *L*. *plantarum*-mCherry and maintained on fresh food for the remainder of the experiment (Fig 4A). In a parallel cohort of homogenized flies, measurements of colony-forming units (CFUs) confirmed that levels of *L*. *plantarum* were high immediately after the 1-day pulse, decreased over the next 3 days, and subsequently plateaued (Fig 4B), indicating stable colonization [24].

To reveal the bacteria’s spatial distribution within single animals over time, we performed serial, whole-abdomen Bellymount imaging at 0, 2, and 4 days after the *L*. *plantarum* pulse (Fig 4C–4G). At the 0-day time point, *L*. *plantarum*-mCherry densely occupied the lumens of both the crop (a proximal storage organ [25]) and the midgut, with a filling fraction of 51 ± 15% (*n* = 9) (Fig 4D and 4G). At the 4-day time point, *L*. *plantarum*-mCherry was sparse in both organs, with a filling fraction of 4.6 ± 4.7% (*n* = 5) (Fig 4D and 4G). These observations were consistent with CFU measurements (Fig 4B).

Beyond filling fraction, the three-dimensional patterns of *L*. *plantarum* exhibited intriguing regional and temporal dynamics that could not have been detected in homogenized animals. During crop colonization, *L*. *plantarum* localized to the crop wall, where it frequently coalesced into prominent clumps (*n* = 4) (Fig 4D and 4E and S8 Movie). By contrast, during midgut colonization, *L*. *plantarum* remained in the lumen (S6 Fig). In the lumen of the proximal R2 midgut region (S7 Fig), *L*. *plantarum* coalesced into clumps (*n* = 3) (Fig 4G and S9 Movie), while in the lumen of the distal R5 region (S7 Fig), the bacteria were sparsely dispersed as single cells (*n* = 1) (Fig 4G and S10 Movie). These regional differences are consistent with impaired bacterial viability after transit through the acidified lumen of the middle midgut [26]. Altogether, this time-resolved analysis of *L*. *plantarum* colonization provides the first insights into the dynamic regional biogeography of the *Drosophila* gut microbiota (Fig 4H).

## Discussion

In summary, Bellymount enables longitudinal studies in the *Drosophila* abdomen through serial, submicron-resolution imaging of animals that are live, intact, and viable. Using the Bellymount platform, we observed real-time digestive transit, visualized the native arrangement of abdominal organs, and captured high-resolution, volumetric images of the organs’ constituent cells and resident microbiota. We applied serial Bellymount imaging to perform time-resolved tracking of midgut stem cell lineage dynamics and gut bacterial colonization, two multiday processes whose precise temporal dynamics were previously inaccessible. These experiments revealed previously undescribed heterogeneities in the cellular events that underlie midgut physiology and host–microbiota interactions.

In addition to its unique scientific capabilities, the Bellymount platform is inexpensive to implement, versatile in application, and compatible with a wide range of upright and inverted microscope systems. Its simple design, using Elmer’s glue and a 3D-printed apparatus, make the protocol easily accessible to a broad variety of researchers for study of real-time, in vivo dynamics of diverse cellular, physiological, and pathological processes in adult *Drosophila*.

## Materials and methods

### *Drosophila* stocks

We obtained *hemolectinGal4; UAS-2xEGFP* (BL30140), *ubi-his2av*::*mRFP* (BL23650), *UAS-LifeActGFP* (BL35544), and *10xUAS-IVS-myr-td*::*Eos* (*UAS-Eos*) (BL32226) from the Bloomington Stock Center. *esgGal4* (112304) was obtained from the Kyoto Drosophila Genomics and Genetics Resource (DGGR). The following stocks were gifts: *mexGal4* (Carl Thummel), *breathlessGal4*, *UAS-his2b*::*CFP* (Yoshihiro Inoue), *ubi-his2avD*::*YFP* (Pavel Tomancak), and *GBE-Su(H)-GFP*::*nls* (Joaquin de Navascues), *UAS-CD8-GFP*, *hs-flp*^*12*^*; tubGal4; FRT82*, *tubGal80* and *FRT82* (David Bilder). The “fate sensor” line (*esgGal4*, *UAS-his2b*::*CFP*, *GBE-Su(H)-GFP*::*nls; ubi-his2av*::*mRFP*) was generated previously [12].

### *Drosophila* husbandry

All experiments were performed on adult females. Flies and crosses were kept at 25°C unless otherwise indicated. Animals were raised on standard cornmeal–molasses media (water, molasses, cornmeal, agar, yeast, Tegosept, propionic acid). For experiments, unless otherwise indicated, adult females were kept on cornmeal–molasses vials supplemented with a pinch of powdered dry yeast (Red Star, Active Dry Yeast) for 4 days prior to imaging. Males were included in these vials.

### Fabrication of Bellymount apparatus and humidity chamber

The Bellymount apparatus consists of a lid, base, and humidity chamber (Fig 1D and S2 Fig). The base and lid were 3D-printed using the online service Shapeways (https://shapeways.com). Fabrication was performed with fine detail plastic (Visijet M3 Crystal UV curable plastic; 3D Systems, Rock Hill, SC, USA) and the basic “smooth” finish option.

To prevent the animal from desiccating during imaging, we attached a humidity chamber to the underside of the apparatus base (S2 Fig). Briefly, we drilled a 3-mm outlet for CO_2_ into the wall of a 35-mm petri dish (Olympus plastics, #32–103; Genesee Scientific Corporation, El Cajon, CA, USA), then adhered the chamber to a small groove on the underside of the base using dental wax (Surgident, #50092189; Daegu, Korea). Lastly, we covered the bottom of the petri dish with trimmed paper towels moistened with H_2_O.

### Animal preparation

Flies were glued to the imaging coverslip (Fig 1A and 1B and S1 Movie) as follows: before gluing, individuals were put into a “chill coma” by chilling on ice in a microfuge tube for at least 1 h. A small rectangle of Clear Elmer’s Liquid School Glue (Amazon, B06WVDBR62), roughly the size of the fly abdomen, was painted onto the center of a 40-mm coverslip (Thermo Fisher Scientific, #NC0018778; Waltham, MA, USA) using a Worm Pick (Genesee, #59-AWP [handle] and #59-32P6 [tips]). After applying the glue, flies were quickly adhered to the coverslip.

Gluing the animal on its left ventrolateral surface provided optimal viewing of the GI tract. To achieve the desired positioning, the two most posterior legs of the animal were held with Dumont #5 forceps while laying its ventrolateral side onto the patch of Elmer’s glue. During gluing, care was taken to ensure none of the legs were trapped between the abdomen and the coverslip. After positioning the animal, the abdomen was gently pressed into the Elmer’s glue using a paintbrush.

We found that maximizing contact of the abdominal cuticle with the coverslip maximized visibility of abdominal organs. To achieve this, the abdomen was gently compressed after gluing by placing a second “compressing coverslip” on top atop the animal (Fig 1A and S1 Movie). The compressing coverslip was a square coverslip (Thermo Fisher Scientific, #12-541B) that had been broken in half. For flies fed on yeast powder, we found that 0.48 mm is the optimal distance between the compressing coverslip and the primary coverslip to ensure that the animal experienced compression without undue force (for starved flies, see “Starvation” below). To position the compressing coverslip, two adherent spacers (Millipore-Sigma, #GBL620004-1EA; Burlington, MA, USA) were placed on either side of the animal. For well-fed females, spacers of 0.48-mm thickness were used; for starved females (cf. S8 Fig), spacers of 0.24-mm thickness were used. The compressing coverslip was laid on top of the spacers. After securing the animal thusly, the coverslip was nested inside the base of the apparatus, and the lid was screwed on.

### Anesthesia during Bellymount imaging

To minimize voluntary and involuntary tissue movements, we applied CO_2_ anesthesia during imaging. CO_2_ was delivered using 2-mm inner diameter (ID) flexible silicone tubing attached to the inlet built into the base of the Bellymount apparatus (Fig 1D). To prevent desiccation of the animal, the tubing was connected to a 500-mL PeCon humidification bottle (PeCon, Erbach, Germany) containing distilled water. The humidified CO_2_ was piped through a secondary regulator (Micromatic, #8011–15; Brooksville, FL, USA), which allowed fine control of CO_2_ flow during imaging. The secondary regulator was attached, in turn, to a primary regulator and CO_2_ tank.

### Release of Bellymounted animals

To release animals from the Bellymount apparatus after imaging, the compressing coverslip and spacers were removed as a single unit and saved for future experiments. The tip of a Dumont #5 forceps was placed under the thorax to gently pry the animal from the dried Elmer’s glue (S2 Movie).

Occasionally, a layer of Elmer’s glue remained on the fly's abdomen after it was removed. This layer could be peeled off easily by grabbing a free tab of dried glue with a pair of forceps. If no free surface of dry glue was present, the glue was rewetted with milliQ H_2_O using a small paintbrush. After allowing the rewetted glue to dry, a free tab of Elmer’s glue would commonly present itself. The glue was then peeled off with forceps as described above.

### Survival assay

To test for potential effects of the Bellymount protocol on viability, we measured the lifespans of female flies glued to a coverslip and compressed (see “Animal preparation”). Adult females were collected immediately after eclosion and placed in molasses vials with powdered dry yeast and males for 4 days before the experiment. Animals were then randomly split into two groups (Bellymounted, *n* = 50; control, *n* = 49). Bellymounted animals were glued and compressed as described above (see “Animal preparation”), except that animals were held at room temperature in an empty pipette tip box with moistened paper towels rather than in the imaging apparatus. After 1 h, animals were released from the Bellymount apparatus as described above (see “Release of Bellymounted animals”) and placed in a vial with 8–10 other experimental females and 3–4 males. The control group, which was not subjected to the Bellymount protocol, was maintained under identical conditions. We recorded the number of deceased individuals each day and flipped the remaining survivors onto fresh food. Flies were maintained at 25°C over the course of the experiment.

### Microscopy

We collected confocal images and data using 4 microscope systems: (1) an inverted Zeiss LSM880 with Zen software and Airy scan mode (Carl Zeiss, Oberkochen, Germany) (Figs 2C–2E and 4C–4D, and S7 Fig); (2) an inverted Zeiss LSM780 microscope with Zen software (Figs 2H, 4E–4G, S6 Fig, S6 Movie, and S8–S10 Movies); (3) an upright Leica SP5 confocal (Leica, Wetzlar, Germany) (Figs 1F, 2A, 2B, 2F, 2G, 3B, 3D, 3G and 3J; S3, S4, and S8 Fig; and S4, S5 and S7 Movies); and (4) an inverted Leica SP8 confocal (S5 Fig). To preserve fly viability, imaging was performed with the shortest exposure time and lowest laser power that allowed for acquisition of high-quality images.

We acquired brightfield images using 3 microscope setups: (1) a Zeiss Discovery V8 stereodissection microscope coupled with an iPhone 5S camera (Apple, Cupertino, CA, USA) (S2 Movie); (2) a Leica stereodissection microscope coupled with an iPhone X camera (Fig 1B); (3) a Leica stereodissection microscope coupled with an iPhone 5S camera (S1 Movie); and (4) a digital, USB-pluggable microscope (Plugable.com, USB2-MICRO-250X Digital Microscope; Redmond, WA, USA) (Fig 1C and S3 Movie).

Inverted and upright setups used the identical Bellymount apparatus, which was positioned so that the imaging coverslip faced the objective. Specific imaging parameters were determined for each experiment, depending on microscope setup, imaging objective, fluorescent marker expression, and other factors. In general, flies were exposed to laser light and CO_2_ for no more than 30 min.

### Determining visible regions of the midgut

To determine which regions of the midgut are visualized by Bellymount imaging, we used Eos, a photoconvertible fluorophore [27], to mark light-exposed regions of the midgut tube. Four-day–old animals (*mexGal4*, *Gal80*^*ts*^*; ubi-his2av*::*mRFP/UAS-Eos*) were glued and prepared as described above (see “Animal preparation”). Using 405-nm laser light on a Zeiss LSM880 confocal, Eos protein was photoconverted from green to red emission.

After photoconversion, animals were removed from the Bellymount apparatus (see “Release of Bellymounted animals”), and the midgut was examined ex vivo to identify the photoconverted regions. Specifically, an 8-well Secure-Seal spacer sticker (Thermo Fisher Scientific, #S24737) was used to form “wells” on a microscope slide (Thermo Fisher Electronic Microscopy Sciences, #63720–05). After dissecting in Schneider’s insect medium (Sigma-Aldrich, #S0146; St. Louis, MO, USA), an individual midgut and 7 μL of Schneider’s medium were placed in each well. The wells were topped with a coverslip (Thermo Fisher Scientific, #12-545-81). Midguts were imaged using an inverted Zeiss LSM 880 immediately after mounting.

### Time-lapse imaging of nutrient ingestion and midgut peristalsis

To monitor nutrient ingestion and midgut peristalsis, we performed low-magnification imaging of the fly abdomen during feeding of a dye-colored liquid nutrient solution. Animals were mounted as described above (see “Animal preparation”). A cotton feeding wick was positioned in proximity to the fly’s proboscis and glued to the coverslip using KWIK-SIL silicone glue (World Precision Instruments, 60002; Sarasota, FL, USA). The feeding wick was attached to 2-mm ID flexible silicone tubing connected to a 10-mL syringe reservoir (Thermo Fisher Scientific, #03 377 23) and filled with 10% Brilliant Blue FCF Dye in 5% sucrose (Sigma-Aldrich, #84097) water. Once the feeding wick was positioned and filled, the coverslip was gently placed into the apparatus with the tubing, and the reservoir was attached through the CO_2_ outlet.

For imaging, a USB-pluggable microscope (Plugable.com, USB2-MICRO-250X Digital Microscope) was positioned above the abdomen of the fly. Images were acquired at 2 frames s^−1^.

### Longitudinal imaging, tracking, and analysis of stem cell clones with MARCM

The MARCM system [15] was used to generate GFP-marked stem cell clones. MARCM enables permanent, heritable GFP expression specifically in a subset of mitotic cells for which chromosomal recombination results in loss of GAL80^ts^ and consequent tubGAL4-driven expression of UAS-GFP in one daughter. In the adult fly midgut, MARCM specifically labels stem cell lineages because stem cells are, with rare exceptions, the only cell type that undergoes mitosis [16,18,28].

Crosses for MARCM labeling were maintained at 18°C prior to eclosion and shifted to 25°C within the first 8 h after eclosion. In our hands, this temperature shift resulted in spontaneous GFP labeling of a small fraction of midgut stem cells. Four days after the 25°C temperature shift, we performed the first session of Bellymount imaging (day 0) (Fig 3A and S4 Fig). The sparseness of these spontaneous clones facilitated reidentification of clones during subsequent imaging sessions. After each imaging session, animals were placed in fresh vials (1 animal/vial) with powdered dry yeast and one male at 25°C. Animals were flipped to new vials each day.

We identified and analyzed midgut MARCM clones by examining serial confocal sections. Only clones that could be unambiguously reidentified for 3 consecutive time points were analyzed. Clone identification involved comparing the clone's position within the gut tube to that of neighboring clones (S4 Fig). In some cases, clones had a distinctive shape that allowed them to be reliably identified across imaging sessions. Of midguts imaged for two or more time points, 43% (9/21 midguts) had at least one trackable clone.

MARCM clones were analyzed using FIJI and Bitplane Imaris v. 9.3.0. Cells per clone were determined by counting the number of contiguous cells in a discrete clone labeled with the nuclear marker *ubi-his2av*::*mRFP*. Nuclear volumes were determined using the Imaris contour tool to create a surface from each *ubi-his2av*::*mRFP*-labeled nucleus and measuring the enclosed volume.

### Determination of characteristic nuclear volumes for midgut cell types

To establish characteristic nuclear volumes of stem cells, enteroblasts, and enterocytes for Bellymount clone analyses (Fig 3E, 3H and 3K), we measured nuclear volumes of these 3 cell types in fixed midguts (S5 Fig). Animals of genotype *esgGal4*, *UAS-his2b*::*CFP*, *GBE-Su(H)-GFP*::*nls; ubi-his2av*::*mRFP* were fixed, immunostained, and mounted as described previously [28]. Primary antibody: mouse anti-Prospero (1:400, DSHB, #MR1A), which stains enteroendocrine cells. Secondary antibody: Alexa Fluor 647-conjugated goat anti-mouse IgG (1:400, Thermo Fisher Scientific #A-21240). Samples were mounted in ProLong (Life Technologies, Carlsbad, CA, USA). Stem cells, enteroblasts, and enterocytes were identified as described previously [12]. Nuclear volumes were measured as described above (see “Longitudinal imaging, tracking, and analysis of MARCM clones”).

### *L*. *plantarum* colonization

For experiments with *L*. *plantarum*, flies were raised on standard cornmeal–molasses food. Beginning with *L*. *plantarum* feeding and for the duration of the experiment, flies were shifted to cornmeal–molasses food lacking Tegosept and supplemented with 10 μg/mL chloramphenicol (Calbiochem 220551; Sigma-Aldrich).

A wild fly isolate of *L*. *plantarum* was tagged with a plasmid encoding mCherry and chloramphenicol resistance [24]. This strain was grown overnight from a frozen stock (25% glycerol) in 3 mL MRS medium (Difco Lactobacilli MRS Broth, BD #288110; Thermo Fisher Scientific) containing 10 μg/mL chloramphenicol. We spun down 1.5 mL of the saturated culture at 2,000 rpm for 5 min at 4°C. Next, we resuspended the pellet in 200 μL MRS + 10 μg/mL chloramphenicol and pipetted it onto Whatman paper (Sigma-Aldrich, #WHA1002110) on fresh cornmeal–molasses medium. Ten females and 3 males were transferred to each vial, which was wrapped in aluminum foil to prevent photobleaching of bacteria. Vials were placed at 29°C for 24 h to induce rapid bacterial growth. After 24 h, we removed flies either for imaging or for CFU measurements (see “CFU measurements”). Flies kept for longitudinal imaging were placed in fresh vials at 25°C with 1 male and flipped each day onto fresh food.

### CFU measurements

To quantify bacterial loads, we used 3 flies per day kept in the same conditions as animals used for Bellymount imaging. Flies were kept on ice for 2–4 h and then washed with 70% ethanol 3 times and sterile PBS 3 times to remove external bacteria. Animals were homogenized individually in 1.5-mL microcentrifuge tubes containing 100 μL sterile PBS using a motorized pestle. Next, the homogenate was diluted 10 times 1:5 in sterile PBS, and 3 μL was spotted onto MRS agar plates (1.5% agar) containing 10 μg/mL chloramphenicol. Colonies were counted after growing for 48 h at 30°C.

### Analysis of *L*. *plantarum* filling fraction

To quantify the bacterial density in the digestive tract following fly feeding, we measured the filling fractions of the crop and midgut. Images were analyzed using custom MATLAB R2018b (The MathWorks, Natick, MA, USA) code. To calculate the filling fraction, the edge of either the crop or the midgut was identified from the bacterial signal. The filling fraction was calculated as the pixel area occupied by cells approximately 5 μm into the organ in the *z*-direction using a manually selected intensity threshold to capture signal from bacteria.

### Starvation

To image starved flies, 7- to 10-day–old adult female flies were kept with males on a flug saturated with water for 2.5 days. Flies were swapped to new vials with water-soaked flugs each day. Prior to imaging, flies were glued as described above (see “Animal preparation”). To ensure proper compression, the distance between the compressing and primary coverslips was reduced from 0.48 mm to 0.24 mm (S8 Fig).

## Supporting information

S1 Fig Longevity of animals after Bellymount. Lifespans of Bellymounted females (*n* = 50) were compared to a control cohort of age- and sex-matched animals that were not subjected to Bellymount (*n* = 49). One day after being released, 92% of Bellymounted animals were alive. Genotype: *ubi-his2avD*::*YFP*. The data underlying this figure are included in S3 Data. We used this empirically determined 92% survival rate following one Bellymount session to calculate a theoretical survival rate after 3 sessions of 78% (0.92^3^ = 0.78). This calculation assumes that each session has an equal effect on individual mortality and does not take into account other factors that affect survival, including handling skill, exposure to laser light, and exposure to CO_2_. *his2av*, histone variant His2av; *YFP*, yellow fluorescent protein.
(TIF)

S2 Fig Humidity chamber. To prevent the animal from desiccating, a humidity chamber (35-mm petri dish containing H_2_O-soaked Kimwipes) was used in conjunction with the Bellymount apparatus. The chamber attaches to a groove in the underside of the apparatus base.
(TIF)

S3 Fig Visualization of actin cytoskeletal filaments using Bellymount. To demonstrate Bellymount’s ability to visualize subcellular structures, we examined the actin cytoskeleton (LifeActGFP, grayscale) of immature diploid cells (stem cells and enteroblasts) in the midgut epithelium. Immature cells displayed diverse actin cytoskeletal morphologies. In the panel shown, the pair at top right exhibits pronounced cortical filaments and a few bright puncta, whereas the cell in the middle exhibits weak cortical filaments and numerous dimmer puncta. Magenta (*his2av*::*RFP*) labels all nuclei. Genotype: *esg>LifeActGFP; ubi-his2av*::*mRFP*. This experiment used 7- to 10-day–old adult females that were fed on cornmeal–molasses food supplemented with dry yeast powder for 2 days. *esg*, *escargot-*Gal4; *GFP*, green fluorescent protein; *his2av*, histone variant His2av; *mRFP*, monomeric red fluorescent protein; *RFP*, red fluorescent protein.
(TIF)

S4 Fig Persistent organ-level patterns enable reidentification of midgut clones. Wide-field view of the midgut of Fly 3 at each imaging time point (Fig 3A and 3B). GFP-labeled stem cell clones were visible as green multicellular clusters. Blue boxes outline trackable clones that were analyzed in detail (Fig 3D–3L). Some spontaneous clones (orange arrowheads) appeared over the duration of the experiment. Grayscale, nuclei. *GFP*, green fluorescent protein.
(TIF)

S5 Fig Enterocyte differentiation is characterized by increased nuclear volume. To determine characteristic nuclear volumes of midgut cell types, we used midguts that expressed cellular markers for successive stages of enterocyte differentiation: stem (and stem-like) cells, immature enteroblasts, and mature enterocytes. Midguts were fixed and subjected to volumetric confocal imaging. Volumetric reconstructions were used to measure nuclear volumes for each cell type (mean ± SD): stem cells (*n* = 12), 71.8 ± 16.8 μm^3^; enteroblasts (*n* = 14), 174.2 ± 93.1 μm^3^; enterocytes (*n* = 12), 378.7 ± 139.3 μm^3^. Genotype: *esg>his2b*::*CFP*, *GBE-Su(H)-GFP*::*nls; ubi-his2av*::*mRFP*. The data underlying this figure are included in S4 Data. *CFP*, cyan fluorescent protein; *esg*, *escargot*-Gal4; *GBE*, Grainyhead binding element; *GFP*, green fluorescent protein; *his2av*, histone variant His2av; *mRFP*, monomeric red fluorescent protein; *nls*, nuclear localization sequence; *RFP*, red fluorescent protein; *Su(H)*, Suppressor of Hairless.
(TIF)

S6 Fig *L*. *plantarum* occupies the lumenal space of the midgut during colonization. Planar and orthogonal views of *L*. *plantarum*-mCherry (inverted grayscale) in the proximal (R2) (A) and distal (R5) regions (B) of the midgut were taken 2 days after a *L*. *plantarum* pulse. Planar views (top panels) panels are the same as in Fig 4G. Dotted magenta lines in planar views indicate the slices depicted in the ortho views (bottom panels). Dotted magenta lines in ortho views denote the lumenal surface of the midgut tube, as estimated by visual inspection. *L*. *plantarum*-mCherry (blue arrowheads) occupied the lumenal space of the midgut and did not preferentially localize to the lumenal wall. See S9 and S10 Movies. Genotype for all panels: *ubi-his2avD*::*YFP*. *his2av*, histone variant His2av; *YFP*, yellow fluorescent protein.
(TIF)

S7 Fig Regions of the midgut visualized by Bellymount. To determine which midgut regions are visible by Bellymount, we used animals with midgut-specific expression of Eos, a green-to-red photoconvertible fluorophore (*mex>Eos*). During Bellymount imaging, Eos was photoconverted in visible regions of the midgut. These regions were subsequently identified after dissection and examination ex vivo. (A) Whole abdomen of Bellymounted animal before photoconversion. The two visible midgut loops (white box) exhibited green Eos fluorescence. (B) Midgut after photoconversion. Image is a close-up of boxed area in A. Photoconverted regions (dotted outlines) exhibited red Eos fluorescence (magenta pseudocolor). Nonconverted areas remained green. One region was photoconverted in the ventral loop (orange dotted outline), and two regions were photoconverted in the dorsal loop (white dotted outlines). (C,D) Comparison of midgut after dissection (C) to stereotyped anatomy of midgut regions (D) enables identification of the photoconverted regions. The ventral loop is part of R2, and the dorsal loop is part of R5. All animals examined (11/11) exhibited the same pattern of photoconversion. Weak photoconversion was also apparent in an area of R4 (arrow) that contacts R2 in situ. *mex*, mex1-Gal4.
(TIF)

S8 Fig Comparative anatomy of fed and starved female abdominal organs. Whole-abdomen images of fed (A) and starved (B) females were acquired using Bellymount. The midgut (orange overlay) and ovaries (pink overlay) were markedly smaller and the abdomen was shorter in the starved female. Panel (A) is the same as Fig 2A and shows a 4-day–old, mated adult female fed on standard cornmeal–molasses food with yeast powder. Panel (B) shows a 2.5-day–old, mated female that was fed only water post eclosion. To accommodate the starved animals’ smaller size during imaging, the height of the spacers between the imaging and compression coverslips was reduced by half (from 0.48 mm to 0.24 mm). Genotype: *esg>LifeActGFP; ubi-his2av*::*mRFP* (only RFP is shown). Grayscale, nuclei. *esg*, *escargot*-Gal4; *GFP*, green fluorescent protein; *his2av*, histone variant His2av; *mRFP*, monomeric red fluorescent protein; *RFP*, red fluorescent protein.
(TIF)

S1 Movie Video tutorial of Bellymount gluing protocol. (MP4)

S2 Movie Release of Bellymounted animal after imaging. Gentle prying with forceps removed a Bellymounted animal from Elmer’s glue on the imaging coverslip. The animal was intact and immediately walked out of view.
(MP4)

S3 Movie Real-time nutrient ingestion, GI transit, and intestinal peristalsis. A Bellymounted animal was provided a cloth wick saturated with 5% sucrose water colored by Brilliant Blue FCF. Over the 45-min imaging session, the ingested liquid filled the crop and successive compartments of the midgut. Rapid peristaltic contractions of the midgut tube were visible. GI, gastrointestinal.
(MP4)

S4 Movie Carbon dioxide (CO2) anesthesia inhibits tissue movement during confocal imaging. Time-lapse confocal imaging of the midgut in a Bellymounted animal. Continuous imaging was performed while CO_2_ flow through the Bellymount apparatus was toggled on and off. With CO_2_ off, tissue movement caused individual cells to be captured multiple times. With CO_2_ on, tissue movement was inhibited, and no repeated images were observed. Red, all nuclei; green, midgut stem cells and enteroblasts. Time intervals for CO_2_ off and on were 15 min and 20 min, respectively. Each movie frame is a *z*-stack projection. Forty-one *z*-stacks were captured at 5-min intervals. Each stack required approximately 2 min to acquire 25 optical sections at intervals of 3 μm. Total elapsed time was 3.4 h. Genotype: *esg>LifeActGFP; ubi-his2av*::*mRFP*. *esg*, *escargot*-Gal4; *GFP*, green fluorescent protein; *his2av*, histone variant His2av; *mRFP*, monomeric red fluorescent protein; *RFP*, red fluorescent protein.
(AVI)

S5 Movie Native arrangement of female abdominal organs at high resolution. Animated *z*-stack of the tiled, whole-abdomen projection shown in Fig 2A. Single optical sections through Bellymounted female were taken at 3-μm steps from the exterior cuticle to an interior depth of 54 μm. All nuclei are marked with *his2av*::*mRFP* (inverted grayscale). Scale is as indicated in Fig 2A. *his2av*, histone variant His2av; *mRFP*, monomeric red fluorescent protein; *RFP*, red fluorescent protein.
(AVI)

S6 Movie Volumetric image of developing egg chambers. Three-dimensional reconstruction of egg chambers in the ovary of a Bellymounted female. Nascent oocytes and supporting nurse and follicle cells were readily identifiable. Labels indicate eggs at different developmental stages, the surrounding fat body, and adjacent cuticle. All nuclei are marked with *his2av*::*mRFP* (grayscale). Scale bar, 30 μm. *his2av*, histone variant His2av; *mRFP*, monomeric red fluorescent protein; *RFP*, red fluorescent protein.
(MP4)

S7 Movie *Z*-stack animation of 6-day MARCM clone in Fig 3G. Animation shows the 32 optical sections of the complete, original z-stack containing the 6-day, GFP-marked clone. Sections progress from the exterior cuticle to the midgut lumen at 1 μm intervals. Each of the 10 nuclei within the clone are numbered when they first appear in the animation. Nuclei in the animation are pseudocolored magenta, and scale is same as in Fig 3G. *GFP*, green fluorescent protein; MARCM, Mosaic Analysis with a Repressible Cell Marker.
(AVI)

S8 Movie Volumetric image of the crop lumen 2 days after colonization with *L*. *plantarum*-mCherry. Volumetric reconstruction of same field as in Fig 4E showing the exterior cuticle and crop. *L*. *plantarum*-mCherry cells lined the edge of the crop lumen and also formed clumps. Round yeast cells were visible in the crop lumen. Grayscale, *L*. *plantarum*-mCherry and autofluorescence of yeast cells and the external cuticle. Scale bar, 10 μm.
(MP4)

S9 Movie Volumetric image of the proximal midgut (R2) 2 days after colonization with *L*. *plantarum*-mCherry. Volumetric reconstruction of the same field as in Fig 4G. Clumps of *L*. *plantarum*-mCherry cells as well as free-floating bacteria were visible in the midgut lumenal space. Yellow dotted lines mark the lumen boundary. Grayscale, *L*. *plantarum*-mCherry. Scale bar, 15 μm.
(MP4)

S10 Movie Volumetric image of the distal midgut (R5) 2 days after colonization with L. plantarum-mCherry. Volumetric reconstruction of the same field as in Fig 4G. Individual, free-floating bacteria were visible within the lumenal space; no clumps were apparent. Yellow dotted lines mark the lumen boundary. Grayscale, *L*. *plantarum*-mCherry. Scale bar, 15 μm.
(MP4)

S1 Data Raw data of Fig 3C, 3E, 3H and 3K. (XLSX)

S2 Data Raw data of Fig 4B. (XLSX)

S3 Data Raw data of S1 Fig. (XLSX)

S4 Data Raw data of S5 Fig. (XLSX)

S1 File CAD file for 3D printing of the Bellymount apparatus lid. CAD, computer-aided design.
(STL)

S2 File CAD file for 3D printing of the Bellymount apparatus base. CAD, computer-aided design.
(STL)

## Abbreviations

CAD: computer-aided design
*CD8*: membrane protein CD8
*CFP*: cyan fluorescent protein
CFU: colony-forming unit
DGGR: Drosophila Genomics and Genetics Resource
DGRC: Drosophila Genomics Resource Center
*esg*: *escargot-*Gal4
*FRT*: Flippase Recognition Target
*GBE*: Grainyhead binding element
*GFP*: green fluorescent protein
GI: gastrointestinal
*his2av*: histone variant His2av
*hml*: hemolectin
*hs-flp*: heat-shock flippase
ID: inner diameter
MARCM: Mosaic Analysis with a Repressible Cell Marker
*mRFP*: monomeric red fluorescent protein
*nls*: nuclear localization sequence
*RFP*: red fluorescent protein
*Su(H)*: Suppressor of Hairless
*tub*: αTubulin84b promoter
*UAS*: Upstream Activating Sequence
USB: universal serial bus
