# Supplementary figures and images for: Bellymount enables longitudinal, intravital imaging of abdominal organs and the gut microbiota in adult Drosophila
Source: PLoS Biol. 2020 Jan 27;18(1):e3000567. doi: 10.1371/journal.pbio.3000567 (PMC7004386; doi:10.1371/journal.pbio.3000567)

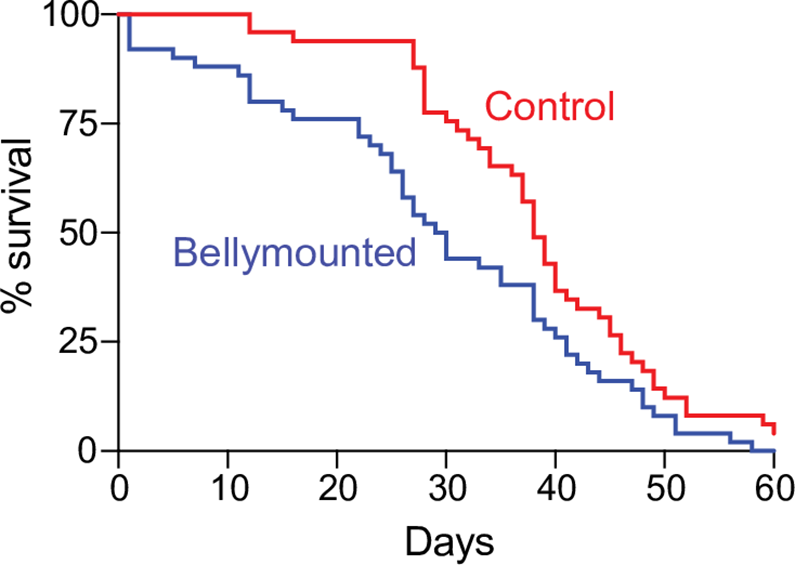

Supplement: S1 Fig — Lifespans of Bellymounted females (n = 50) were compared to a control cohort of age- and sex-matched animals that were not subjected to Bellymount (n = 49). One day after being released, 92% of Bellymounted animals were alive. Genotype: ubi-his2avD::YFP. The data underlying this figure are included in S3 Data. We used this empirically determined 92% survival rate following one Bellymount session to calculate a theoretical survival rate after 3 sessions of 78% (0.923 = 0.78). This calculation assumes that each session has an equal effect on individual mortality and does not take into account other factors that affect survival, including handling skill, exposure to laser light, and exposure to CO2. his2av, histone variant His2av; YFP, yellow fluorescent protein. (TIF) [file pbio.3000567.s001.tif]

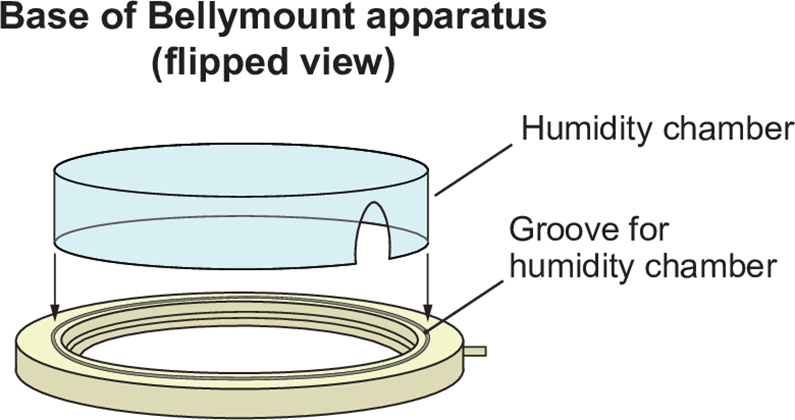

Supplement: S2 Fig — To prevent the animal from desiccating, a humidity chamber (35-mm petri dish containing H2O-soaked Kimwipes) was used in conjunction with the Bellymount apparatus. The chamber attaches to a groove in the underside of the apparatus base. (TIF) [file pbio.3000567.s002.tif]

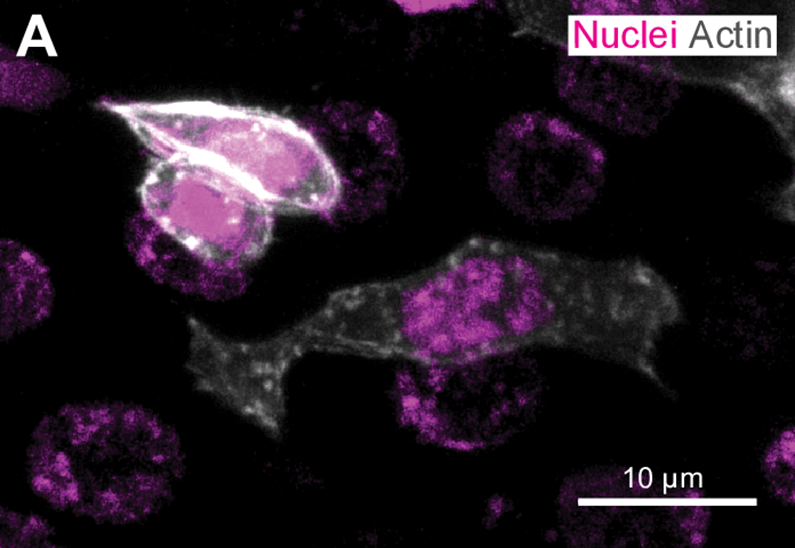

Supplement: S3 Fig — To demonstrate Bellymount’s ability to visualize subcellular structures, we examined the actin cytoskeleton (LifeActGFP, grayscale) of immature diploid cells (stem cells and enteroblasts) in the midgut epithelium. Immature cells displayed diverse actin cytoskeletal morphologies. In the panel shown, the pair at top right exhibits pronounced cortical filaments and a few bright puncta, whereas the cell in the middle exhibits weak cortical filaments and numerous dimmer puncta. Magenta (his2av::RFP) labels all nuclei. Genotype: esg>LifeActGFP; ubi-his2av::mRFP. This experiment used 7- to 10-day–old adult females that were fed on cornmeal–molasses food supplemented with dry yeast powder for 2 days. esg, escargot-Gal4; GFP, green fluorescent protein; his2av, histone variant His2av; mRFP, monomeric red fluorescent protein; RFP, red fluorescent protein. (TIF) [file pbio.3000567.s003.tif]

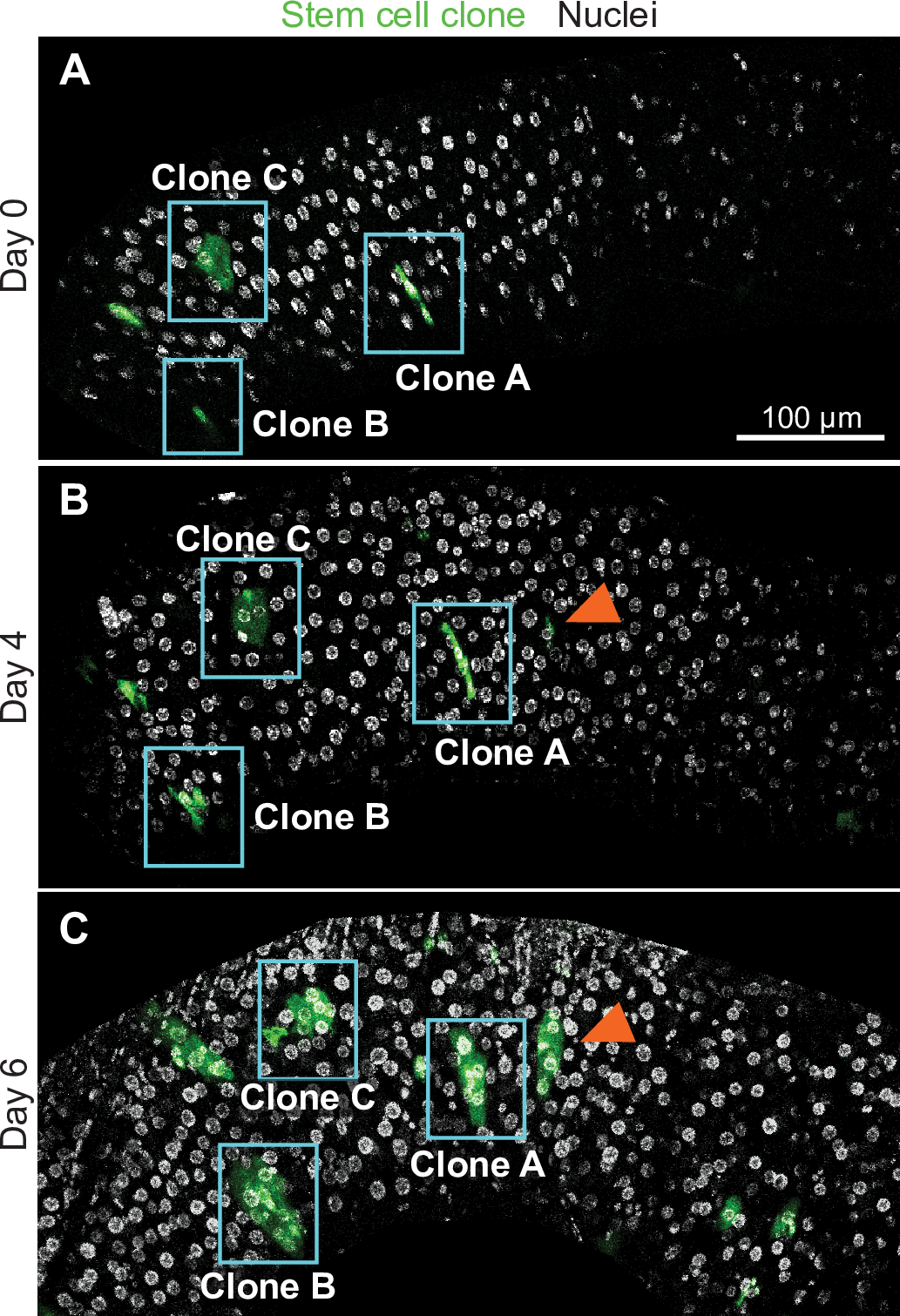

Supplement: S4 Fig — Wide-field view of the midgut of Fly 3 at each imaging time point (Fig 3A and 3B). GFP-labeled stem cell clones were visible as green multicellular clusters. Blue boxes outline trackable clones that were analyzed in detail (Fig 3D–3L). Some spontaneous clones (orange arrowheads) appeared over the duration of the experiment. Grayscale, nuclei. GFP, green fluorescent protein. (TIF) [file pbio.3000567.s004.tif]

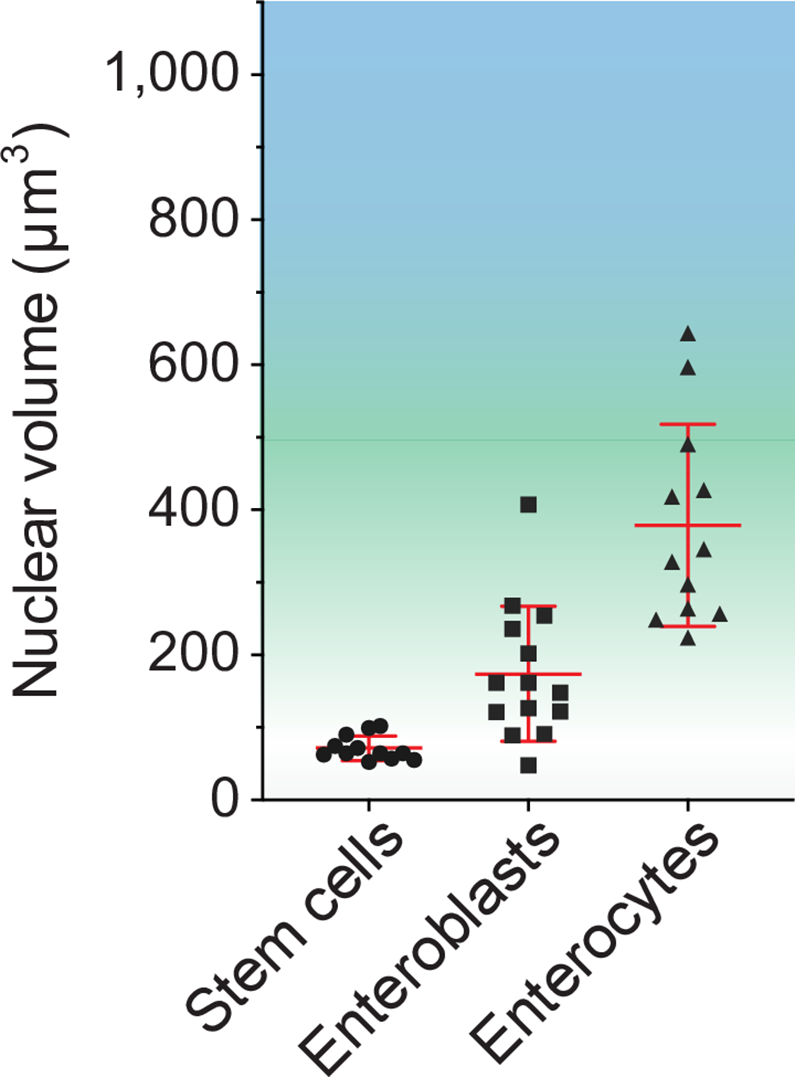

Supplement: S5 Fig — To determine characteristic nuclear volumes of midgut cell types, we used midguts that expressed cellular markers for successive stages of enterocyte differentiation: stem (and stem-like) cells, immature enteroblasts, and mature enterocytes. Midguts were fixed and subjected to volumetric confocal imaging. Volumetric reconstructions were used to measure nuclear volumes for each cell type (mean ± SD): stem cells (n = 12), 71.8 ± 16.8 μm3; enteroblasts (n = 14), 174.2 ± 93.1 μm3; enterocytes (n = 12), 378.7 ± 139.3 μm3. Genotype: esg>his2b::CFP, GBE-Su(H)-GFP::nls; ubi-his2av::mRFP. The data underlying this figure are included in S4 Data. CFP, cyan fluorescent protein; esg, escargot-Gal4; GBE, Grainyhead binding element; GFP, green fluorescent protein; his2av, histone variant His2av; mRFP, monomeric red fluorescent protein; nls, nuclear localization sequence; RFP, red fluorescent protein; Su(H), Suppressor of Hairless. (TIF) [file pbio.3000567.s005.tif]

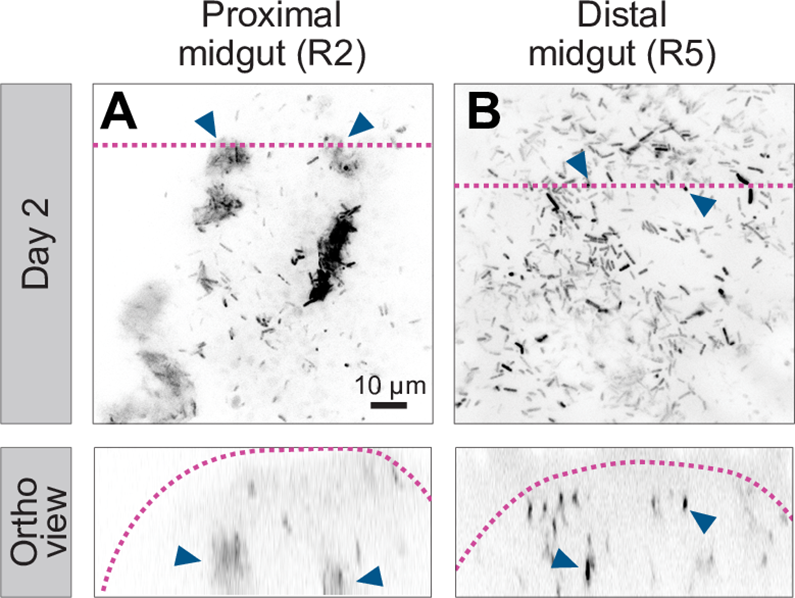

Supplement: S6 Fig — Planar and orthogonal views of L. plantarum-mCherry (inverted grayscale) in the proximal (R2) (A) and distal (R5) regions (B) of the midgut were taken 2 days after a L. plantarum pulse. Planar views (top panels) panels are the same as in Fig 4G. Dotted magenta lines in planar views indicate the slices depicted in the ortho views (bottom panels). Dotted magenta lines in ortho views denote the lumenal surface of the midgut tube, as estimated by visual inspection. L. plantarum-mCherry (blue arrowheads) occupied the lumenal space of the midgut and did not preferentially localize to the lumenal wall. See S9 and S10 Movies. Genotype for all panels: ubi-his2avD::YFP. his2av, histone variant His2av; YFP, yellow fluorescent protein. (TIF) [file pbio.3000567.s006.tif]

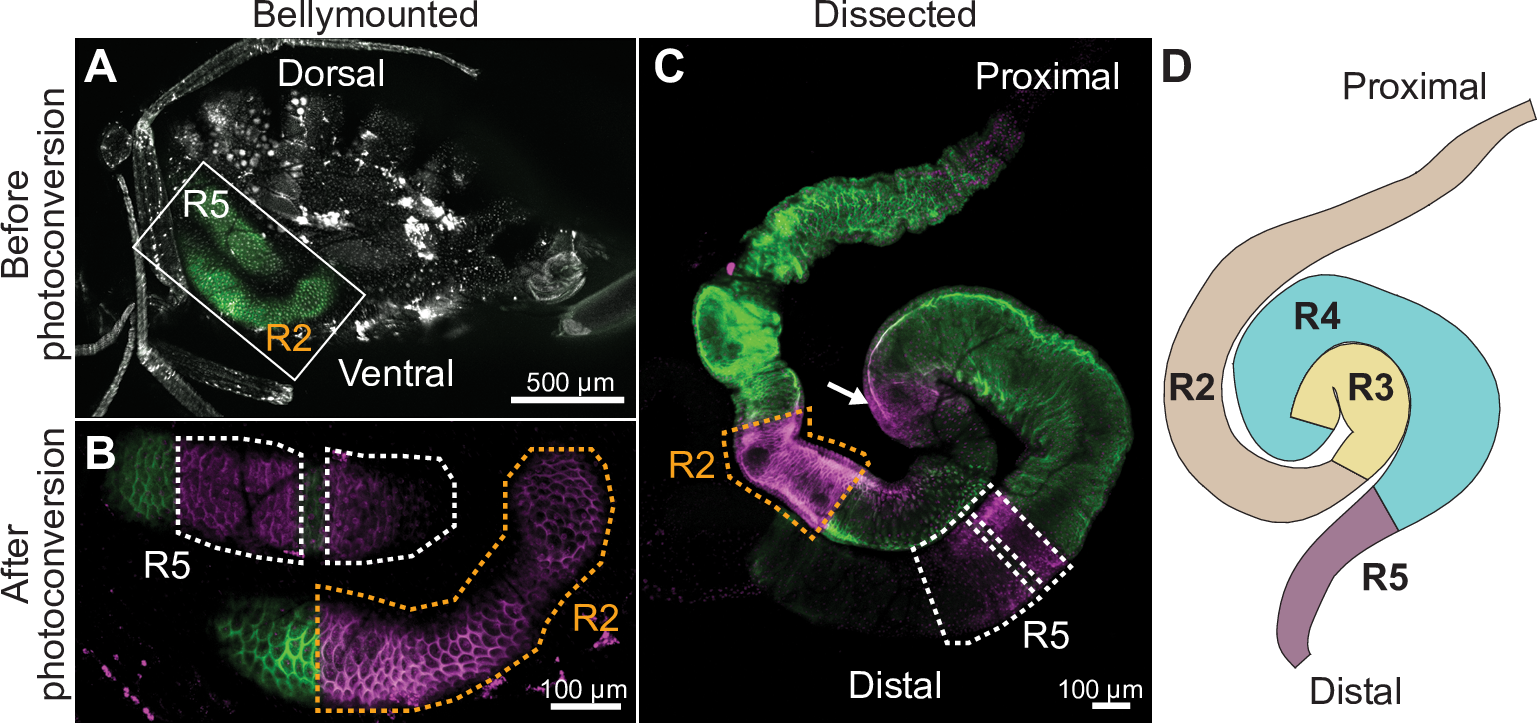

Supplement: S7 Fig — To determine which midgut regions are visible by Bellymount, we used animals with midgut-specific expression of Eos, a green-to-red photoconvertible fluorophore (mex>Eos). During Bellymount imaging, Eos was photoconverted in visible regions of the midgut. These regions were subsequently identified after dissection and examination ex vivo. (A) Whole abdomen of Bellymounted animal before photoconversion. The two visible midgut loops (white box) exhibited green Eos fluorescence. (B) Midgut after photoconversion. Image is a close-up of boxed area in A. Photoconverted regions (dotted outlines) exhibited red Eos fluorescence (magenta pseudocolor). Nonconverted areas remained green. One region was photoconverted in the ventral loop (orange dotted outline), and two regions were photoconverted in the dorsal loop (white dotted outlines). (C,D) Comparison of midgut after dissection (C) to stereotyped anatomy of midgut regions (D) enables identification of the photoconverted regions. The ventral loop is part of R2, and the dorsal loop is part of R5. All animals examined (11/11) exhibited the same pattern of photoconversion. Weak photoconversion was also apparent in an area of R4 (arrow) that contacts R2 in situ. mex, mex1-Gal4. (TIF) [file pbio.3000567.s007.tif]

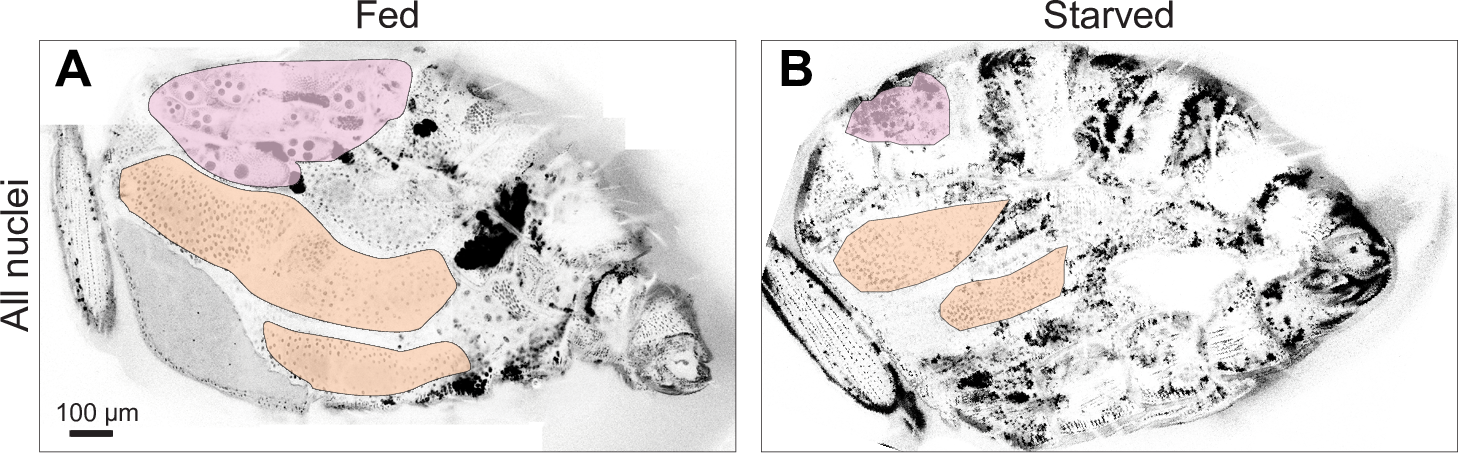

Supplement: S8 Fig — Whole-abdomen images of fed (A) and starved (B) females were acquired using Bellymount. The midgut (orange overlay) and ovaries (pink overlay) were markedly smaller and the abdomen was shorter in the starved female. Panel (A) is the same as Fig 2A and shows a 4-day–old, mated adult female fed on standard cornmeal–molasses food with yeast powder. Panel (B) shows a 2.5-day–old, mated female that was fed only water post eclosion. To accommodate the starved animals’ smaller size during imaging, the height of the spacers between the imaging and compression coverslips was reduced by half (from 0.48 mm to 0.24 mm). Genotype: esg>LifeActGFP; ubi-his2av::mRFP (only RFP is shown). Grayscale, nuclei. esg, escargot-Gal4; GFP, green fluorescent protein; his2av, histone variant His2av; mRFP, monomeric red fluorescent protein; RFP, red fluorescent protein. (TIF) [file pbio.3000567.s008.tif]
