# Supplementary material for: Bellymount enables longitudinal, intravital imaging of abdominal organs and the gut microbiota in adult Drosophila
Source: PLoS Biol. 2020 Jan 27;18(1):e3000567. doi: 10.1371/journal.pbio.3000567 (PMC7004386; doi:10.1371/journal.pbio.3000567)
Supplement: Attachment — Submitted filename: KoyamaLAJ_Rebuttal.docx [file pbio.3000567.s025.docx]

**Reviewer #1**

*It is clearly an exciting method. Its simplicity will allow most labs to use it and it should be of interest to a wide community of Drosophila researchers. The ability to follow a developmental process across several days is a big leap forward. One should be able to study inter-organs communication and fly physiology. The data shown as proof-of-principle are convincing and carefully interpreted. These experiments could not have been done as such with previous methods.*

We thank the Reviewer for these remarks on the data quality and the exciting technical advance that Bellymount represents for longitudinal studies *in vivo*.

*The authors claim that it allows subcellular resolution, but do not show any example. Would it be possible to show microtubules or actin cytoskeleton behavior in the gut using this technique?*

We believe the Reviewer will appreciate new S3 Fig. This Bellymount-acquired image illustrates the subcellular organization of the actin cytoskeleton in midgut progenitor cells (stem cells and enteroblasts). A pair of progenitor cells exhibits strong cortical actin filaments, while a third, lone progenitor cell exhibits weak cortical actin and an abundance of puncta, possibly focal adhesions.

*Does this glue clear other types of insect cuticle? Would it work for notum for example?*

The *Drosophila* ventral abdomen is the only cuticle for which we have attempted the Bellymount protocol. We conjecture that Bellymount’s effectiveness would be reduced, perhaps substantially, by the darker pigmentation and more brittle nature of the dorsal notum (as well as dorsal abdomen). Whether the sternum/ventral notum is amenable to Bellymount is not currently known.

*Is this method applicable beyond Drosophila to other insects or organisms?*

We have not attempted Bellymount with other organisms; we have used the protocol to successfully image *Drosophila* larvae. As we noted above, the success of Bellymount is likely to hinge on the thickness and the pigmentation of the cuticle through which the user is trying to image.

**Reviewer #2**

*In this relatively short but exciting method paper, Leslie et al described a non-invasive method named “Bellymount” that enables long-term imaging of abdominal internal organs in adult Drosophila at single cell resolution. Utilizing this method, the authors traced the growth of intestinal stem cell clones over 6 days, and determined the dynamics of cell addition and differentiation within each ISC lineage. In addition, the authors also successfully utilized this method to examine the dynamics and region preference of gut colonization of fluorescein- labeled commensal bacteria L. plantarum following feeding. This method is relatively simple but may have a lot of potential, as it opens the door to many previously inaccessible biological questions.*

We appreciate the Reviewer’s enthusiasm for the potential impact of Bellymount.

*It would be helpful if the authors can add more details about the imaging procedure, such as the scanning frequency and lasting time at each time point, and any other parameters that may significantly affect the longevity of the animals.*

We performed fluorescent imaging of Bellymounted animals using four different confocal setups to capture diverse types of labelled cells and structures at varying magnifications. Because of this wide variety of conditions, acquisition parameters were tuned on a case-by-case basis to optimize image quality while ensuring that continuous exposure to laser light and CO_2_ remained under 30 minutes. We have observed that viability and longevity of Bellymounted animals become severely impacted when animals are continuously exposed to laser light and CO_2_ for durations that exceed ~30 minutes, likely due to phototoxicity and asphyxiation. This key detail has now been added to the Microscopy section of the Methods.

Brightfield imaging on Bellymounted animals can be performed without CO_2_ (*e.g.* S3 Movie) for multiple hours with no apparent effect on viability.

*The authors claimed that the bacteria are attached to the inner wall in the crop but are dispersed in the lumen of the posterior midgut. Could they provide an orthogonal view of the posterior gut to confirm that the bacteria are not attached to the inner wall in these regions?*

We believe the Reviewer will appreciate new S6 Figure, which provides ortho views of the images shown in Figure 4G. The orthoviews reveal distances of 5-30 µm between *L. plantarum*-mCherry and the gut wall.

*Of course, it would be great if the authors could continue to monitor individual flies for a more extended length of time in either of these two experiments, ideally, until their natural death?*

Continued monitoring of individual flies until their natural death would certainly provide a wealth of fascinating new data. We believe, however, that insights emerging from these data would be ancillary to the main thrust of our techniques paper, which is to report first-of-its-kind methodology for longitudinal analyses of abdominal cells and organs. Monitoring flies throughout their adult lifespan would require a major investment of time and effort, yet not alter our work’s primary contribution. We respectfully suggest that these studies would be most apt for future research focusing on the impacts of organismal ageing.

**Reviewer #3**

*In the manuscript “Bellymount enables longitudinal, intravital imaging of abdominal organs and the gut microbiota in adult Drosophila”, the Authors present a new imaging technique allowing longitudinal studies in the abdomen of Drosophila melanogaster. The Authors first present the setup for Bellymount, then the imaging capabilities in the abdominal cavity at organ level. They then show the ability of capturing MARCM clones in the Drosophila midgut, highlighting their trackability up to 6 days post clonal induction. Finally, they follow bacterial colonization of the gut. Overall, I think that the technique in this manuscript could be a useful resource for the Drosophila community.*

We appreciate the Reviewer’s positive evaluation.

*Changes in nutritional conditions and treatment may greatly influence the size of organs such as the crop (filled, empty) or ovary (mated, non-mated). In turn, this may displace organs such as the gut and, by altering the field of view, make it impossible to longitudinally track clones or other cells of interest, thus greatly reducing the value of the technique. I would like the Authors to assay more in details the robustness of the technique by subjecting the flies to various conditions (starvation and refeeding could be an example used previously by the authors, with or without ovaries could be another example) and report the results accordingly.*

We fully agree with the Reviewer’s point. Some physiological transformations that would benefit immensely from longitudinal analysis also involve global changes in organ size. Our original submission used mated females fed with yeast paste, conditions that produce larger guts and ovaries. Does Bellymount work under conditions that produce smaller organs?

To address this issue, we used Bellymount to image flies under starvation conditions: 2.5-day old, mated adult females that were fed only water post-eclosion. To accommodate the starved animals’ smaller size, the height of the spacers was reduced from 0.48 mm to 0.24 mm. (This information is included the S8 Fig caption and Methods). As expected, the visible ovary and the midgut are dramatically smaller compared to fed animals, and the overall length of the abdomen is substantially reduced. Since image resolution is comparable to fed animals, we expect that tracking individual clones and whole organs during cycles of feeding and starvation will be feasible using Bellymount, but we feel these experiments are beyond the scope of this techniques paper.

*The authors show the ability of tracking clones depending on their shape. We would like to know how often this is possible. Is this a common occurrence or an unlikely event (either due to shape of clones or repositioning of the gut). We would like the Authors to comment and insert data about this aspect and combine it with the previous point.*

We imaged 21 animals containing MARCM clones over two or more timepoints. Of these, 43% (9/21) contained at least one trackable midgut clone, and most of these 9 animals contained more than one trackable clone. This information is now included in the Methods.

*In figure 3 I am excited to see an analysis of the clones, as longitudinal tracking of clone expansion is one of the advantages of Bellymount. However, in some of the clones the drawing reports several nuclei that are not visible in the real image (3G). I would like the Authors to document a bit better what the imaging possibilities are. For instance, they could provide pictures with all the nuclei visible, or show different z stacks.*

We appreciate the Reviewer’s enthusiasm for the clone tracking data. Because the midgut epithelium is pseudostratified, some cell nuclei are obscured in the z-stack projection of the Day 6 clone in Figure 3G. To clarify, we provide an animation that steps through the original, complete z-stack (new S7 Movie). The 32 slices of the stack progress from the exterior cuticle to the midgut lumen at 1 µm intervals. Each of the 10 nuclei that comprise the GFP-marked MARCM clone are numbered when they first appear in the animation.

*One of the main strengths of this technique is the ability to do longitudinal studies. This particular property may greatly enhance the aging field. However, we see clonal analyses only up to 6 days. We would like the Authors to expand the chase to later timepoints (30 days at least). In addition, it is not clear in figure S1 if the population survival after 3 tests at 78% has been checked or it is extrapolated from the 92% survival after one experiment. It would be great if the Authors could perform a longitudinal study chasing clones for a month with more than just 3 Bellymount serial application, and report in addition to the clonal data also the survival.*

We apologize for the confusion regarding the survival calculations in S1 Fig. The value of 78% survival after three tests was not measured empirically. It was extrapolated from the 92% measured survival after one test, assuming three consecutive, independent events (0.92^3^ = 0.78). We have revised the S1 Fig caption to emphasize that 78% is a theoretical maximum that was not empirically measured and that survival rate is influenced by handling skill and by exposure to laser light and CO_2_.

The Reviewer suggests a longitudinal study of clone dynamics that incorporates frequent imaging timepoints over a chase period of at least one month. Such a study would undoubtedly yield an exciting wealth of new data and possibly even provide a first paradigm for the evolution of individual lineages during organismal ageing. In our view, however, generating this kind of dataset is ancillary to the main thrust of our techniques paper, which is to report first-of-its-kind methodology for non-invasive, longitudinal analyses of abdominal cells and organs. We feel that the 6-day clone tracking experiments (Fig 3, S4 Fig, S5 Fig, S7 Movie) and the 4-day bacterial colonization experiments (Fig 4, S6 Fig, S8-S10 Movies) provide ample proof-of-principle for longitudinal tracking. The ageing study suggested by the Reviewer would require a major investment of time and effort, yet not alter our work’s primary contribution. Rather, we respectfully suggest that the proposed experiments are most apt for future research focusing on the impacts of organismal ageing.

*In figure 4 the authors show some data on microbial colonization. We would like to see the repeatability of this observation. Please include in the text the number of repeats for this experiment and include number of flies analyzed and number of repeats and quantification of the phenotype.*

We thank the reviewer for catching this omission. The number of flies analyzed per condition has been added to the results of Fig 4.

*One of the strengths of Bellymount is the possibility to easily adapt this system to microscopical system already in place. However, it is not clear on what type of microscopes (inverted vs upright for example) the arena is employable. Would it be possible for the author to comment and possibly include additional arenas for at least inverted and upright microscopes?*

The Bellymount apparatus is compatible with inverted and upright microscopes; indeed, both were used to collect the data in our study. To switch between inverted and upright systems, the apparatus is simply flipped over. We have added this detail to the Methods and revised the main text to emphasize this versatility.

*Which microscope has been used for what picture is not accurate. For example, Figure 3O and 3P do not exist in the current form. This raises the possibility of complete mismatch also for figures that are currently reported in this list. Please verify.*

We apologize for our error. We have fixed this listing in the Microscopy section of the Methods.

*The video of how to unmount a fly from Bellymount is very useful. However, one crucial point is to be able to mount flies on bellymount in the first place. Please add a video (and illustrations?) on how to install the system, and install the fly.*

We believe the Reviewer will appreciate the video tutorial that is now included as new S1 Movie.
