# Supplementary material for: Bellymount enables longitudinal, intravital imaging of abdominal organs and the gut microbiota in adult Drosophila
Source: PLoS Biol. 2020 Jan 27;18(1):e3000567. doi: 10.1371/journal.pbio.3000567 (PMC7004386; doi:10.1371/journal.pbio.3000567)
Supplement: Peer review history

# Editor report: Decision Letter 0

**DOI:** 10.1371/journal.pbio.3000567.r001

**Authors:** Ines Alvarez-Garcia

23 Sep 2019

Dear Lucy, 

Thank you for submitting your manuscript entitled "Bellymount enables longitudinal, intravital imaging of abdominal organs and the gut microbiota in adult Drosophila" for consideration as a Methods and Resources by PLOS Biology.

Your manuscript has now been evaluated by the PLOS Biology editorial staff as well as by an academic editor with relevant expertise and I am writing to let you know that we would like to send your submission out for external peer review.

However, before we can send your manuscript to reviewers, we need you to complete your submission by providing the metadata that is required for full assessment. To this end, please login to Editorial Manager where you will find the paper in the 'Submissions Needing Revisions' folder on your homepage. Please click 'Revise Submission' from the Action Links and complete all additional questions in the submission questionnaire.

Please re-submit your manuscript within two working days, i.e. by Sep 25 2019 11:59PM.

Login to Editorial Manager here: https://www.editorialmanager.com/pbiology

During resubmission, you will be invited to opt-in to posting your pre-review manuscript as a bioRxiv preprint. Visit http://journals.plos.org/plosbiology/s/preprints for full details. If you consent to posting your current manuscript as a preprint, please upload a single Preprint PDF when you re-submit. 

Once your full submission is complete, your paper will undergo a series of checks in preparation for peer review. Once your manuscript has passed all checks it will be sent out for review. 

Feel free to email us at plosbiology@plos.org if you have any queries relating to your submission.

Kind regards,

Ines

--

Ines Alvarez-Garcia, PhD

Senior Editor

PLOS Biology

Carlyle House, Carlyle Road

Cambridge, CB4 3DN

+44 1223–442810

# Peer review: Decision Letter 1

**DOI:** 10.1371/journal.pbio.3000567.r002

**Authors:** Ines Alvarez-Garcia

30 Oct 2019

Dear Lucy,

Thank you very much for submitting your manuscript "Bellymount enables longitudinal, intravital imaging of abdominal organs and the gut microbiota in adult Drosophila" for consideration as a Methods and Resources by PLOS Biology. As with all papers reviewed by the journal, yours was evaluated by the PLOS Biology editors as well as by an Academic Editor with relevant expertise and by three independent reviewers.

Based on the reviews (attached below), we will probably accept this manuscript for publication, assuming that you will modify the manuscript to address all the points raised by the reviewers. After consulting with the Academic Editor, we do not think Point 4 of Reviewer 3 is essential for publication, but we would welcome any data you might already have in hand. Please also make sure to address the data and other policy-related requests noted at the end of this email.

We expect to receive your revised manuscript within two weeks. Your revisions should address the specific points made by each reviewer. In addition to the remaining revisions and before we will be able to formally accept your manuscript and consider it "in press", we also need to ensure that your article conforms to our guidelines. A member of our team will be in touch shortly with a set of requests. As we can't proceed until these requirements are met, your swift response will help prevent delays to publication.

*Copyediting*

Upon acceptance of your article, your final files will be copyedited and typeset into the final PDF. While you will have an opportunity to review these files as proofs, PLOS will only permit corrections to spelling or significant scientific errors. Therefore, please take this final revision time to assess and make any remaining major changes to your manuscript.

NOTE: If Supporting Information files are included with your article, note that these are not copyedited and will be published as they are submitted. Please ensure that these files are legible and of high quality (at least 300 dpi) in an easily accessible file format. For this reason, please be aware that any references listed in an SI file will not be indexed. For more information, see our Supporting Information guidelines:

https://journals.plos.org/plosbiology/s/supporting-information

*Published Peer Review History*

Please note that you may have the opportunity to make the peer review history publicly available. The record will include editor decision letters (with reviews) and your responses to reviewer comments. If eligible, we will contact you to opt in or out. Please see here for more details:

https://blogs.plos.org/plos/2019/05/plos-journals-now-open-for-published-peer-review/

*Early Version*

Please note that an uncorrected proof of your manuscript will be published online ahead of the final version, unless you opted out when submitting your manuscript. If, for any reason, you do not want an earlier version of your manuscript published online, uncheck the box. Should you, your institution's press office or the journal office choose to press release your paper, you will automatically be opted out of early publication. We ask that you notify us as soon as possible if you or your institution is planning to press release the article.

*Submitting Your Revision*

To submit your revision, please go to https://www.editorialmanager.com/pbiology/ and log in as an Author. Click the link labelled 'Submissions Needing Revision' to find your submission record. Your revised submission must include a cover letter, a Response to Reviewers file that provides a detailed response to the reviewers' comments (if applicable), and a track-changes file indicating any changes that you have made to the manuscript. 

Please do not hesitate to contact me should you have any questions.

Sincerely,

Ines

--

Ines Alvarez-Garcia, PhD

Senior Editor

PLOS Biology

Carlyle House, Carlyle Road

Cambridge, CB4 3DN

+44 1223–442810

------------------------------------------------------------------------

DATA POLICY: IMPORTANT - PLEASE READ

You may be aware of the PLOS Data Policy, which requires that all data be made available without restriction: http://journals.plos.org/plosbiology/s/data-availability. For more information, please also see this editorial: http://dx.doi.org/10.1371/journal.pbio.1001797

Note that we do not require all raw data. Rather, we ask that all individual quantitative observations that underlie the data summarized in the figures and results of your paper be made available in one of the following forms:

1) Supplementary files (e.g., excel). Please ensure that all data files are uploaded as 'Supporting Information' and are invariably referred to (in the manuscript, figure legends, and the Description field when uploading your files) using the following format verbatim: S1 Data, S2 Data, etc. Multiple panels of a single or even several figures can be included as multiple sheets in one excel file that is saved using exactly the following convention: S1_Data.xlsx (using an underscore).

2) Deposition in a publicly available repository. Please also provide the accession code or a reviewer link so that we may view your data before publication. 

Regardless of the method selected, please ensure that you provide the individual numerical values that underlie the summary data displayed in the following figure panels as they are essential for readers to assess your analysis and to reproduce it:

Fig. 3C, E, H, K; Fig. 4B; Fig. S1 and Fig. S4

NOTE: the numerical data provided should include all replicates AND the way in which the plotted mean and errors were derived (it should not present only the mean/average values).

Please also ensure that figure legends in your manuscript include information on where the underlying data can be found, and ensure your supplemental data file/s has a legend.

Please ensure that your Data Statement in the submission system accurately describes WHERE YOUR DATA CAN BE FOUND.

-----------------------------------------------------------

Reviewers’ comments

Rev. 1:

In this manuscript, Dr O’Brien and colleagues describe a novel protocol to image Drosophila internal organs for several days. This method is based on the use of a commercial glue, which both immobilizes the fly and makes the abdomen cuticle transparent. As a proof-of-principle, the authors track the progeny of gut stem cells and the colonization of the gut by associated microbes.

It is clearly an exciting method. Its simplicity will allow most labs to use it and it should be of interest to a wide community of Drosophila researchers. The ability to follow a developmental process across several days is a big leap forward. One should be able to study inter-organs communication and fly physiology. The data shown as proof-of-principle are convincing and carefully interpreted. These experiments could not have been done as such with previous methods.

My two main comments/criticisms:

1) *essential* The authors claim that it allows subcellular resolution, but do not show any example. Would it possible to show microtubules or actin cytoskeleton behavior in the gut using this technique?

2) *not essential* Does this glue clear other types of insect cuticle? Would it work for notum for example?

Is this method applicable beyond Drosophila to other insects or organisms?

Rev. 2:

In this relatively short but exciting method paper, Leslie et al described a non-invasive method named “Bellymount” that enables long-term imaging of abdominal internal organs in adult Drosophila at single cell resolution. Utilizing this method, the authors traced the growth of intestinal stem cell clones over 6 days, and determined the dynamics of cell addition and differentiation within each ISC lineage. In addition, the authors also successfully utilized this method to examine the dynamics and region preference of gut colonization of fluorescein- labeled commensal bacteria L. plantarum following feeding. This method is relatively simple but may have a lot of potential, as it opens the door to many previously inaccessible biological questions.

In general, the paper is well-written and in general the method is described in sufficient detail for others to adopt, I only have a few minor points for authors to consider.

1. It would be helpful if the authors can add more details about the imaging procedure, such as the scanning frequency and lasting time at each time point, and any other parameters that may significantly affect the longevity of the animals.

2. The authors claimed that the bacteria are attached to the inner wall in the crop but are dispersed in the lumen of the posterior midgut. Could they provide an orthogonal view of the posterior gut to confirm that the bacteria are not attached to the inner wall in these regions?

3. Of course it would be great if the authors could continue to monitor individual flies for a far more extended length of time in either of these two experiments, ideally, until their natural death?

Rev. 3:

In the manuscript “Bellymount enables longitudinal, intravital imaging of abdominal organs and the gut microbiota in adult Drosophila”, the Authors present a new imaging technique allowing longitudinal studies in the abdomen of Drosophila melanogaster. The Authors first present the setup for Bellymount, then the imaging capabilities in the abdominal cavity at organ level. They then show the ability of capturing MARCM clones in the Drosophila midgut, highlighting their trackability up to 6 days post clonal induction. Finally, they follow bacterial colonization of the gut. Overall, I think that the technique in this manuscript could be a useful resource for the Drosophila communit. However, I have some important concerns that would need to be resolved before publication in Plos Biology.

Major Comments:

1) Robustness of the technique. Changes in nutritional conditions and treatment may greatly influence the size of organs such as the crop (filled, empty) or ovary (mated, non-mated). In turn, this may displace organs such as the gut and, by altering the field of view, make it impossible to longitudinally track clones or other cells of interest, thus greatly reducing the value of the technique. I would like the Authors to assay more in details the robustness of the technique by subjecting the flies to various conditions (starvation and refeeding could be an example used previously by the authors, with or without ovaries could be another example) and report the results accordingly.

2) Robustness of the technique for lineage tracing. A similar concern is for figure 3, where the authors show the ability of tracking clones depending on their shape. We would like to know how often this is possible. Is this a common occurrence or an unlikely event (either due to shape of clones or repositioning of the gut). We would like the Authors to comment and insert data about this aspect and combine it with the previous point.

3) Comparison of the data with established methods. In figure 3 I am excited to see an analysis of the clones, as longitudinal tracking of clone expansion is one of the advantages of Bellymount. However, in some of the clones the drawing reports several nuclei that are not visible in the real image (3G). I would like the Authors to document a bit better what the imaging possibilities are. For instance, they could provide pictures with all the nuclei visible, or show different z stacks. One thing that would be very convincing would be to include the same region imaged after dissection at the end of a time course to match Bellymount with an established “ex-vivo” dissection technique.

4) Length of longitudinal studies. One of the main strengths of this technique is the ability to do longitudinal studies. This particular property may greatly enhance the aging field. However, we see clonal analyses only up to 6 days. We would like the Authors to expand the chase to later timepoints (30 days at least). In addition, it is not clear in figure S1 if the population survival after 3 tests at 78% has been checked or it is extrapolated from the 92% survival after one experiment. It would be great if the Authors could perform a longitudinal study chasing clones for a month with more than just 3 Bellymount serial application, and report in addition to the clonal data also the survival.

5) Repeatability of the findings. In figure 4 the authors show some data on microbial colonization. We would like to see the repeatability of this observation. Please include in the text the number of repeats for this experiment and include number of flies analyzed and number of repeats and quantification of the phenotype.

6) Adaptability of the system. One of the strengths of Bellymount is the possibility to easily adapt this system to microscopical system already in place. However, it is not clear on what type of microscopes (inverted vs upright for example) the arena is employable. Would it be possible for the author to comment and possibly include additional arenas for at least inverted and upright microscopes?

Minor Comments:

- Line 304: which microscope has been used for what picture is not accurate. For example, Figure 3O and 3P do not exist in the current form. This raises the possibility of complete mismatch also for figures that are currently reported in this list. Please verify.

- The video of how to unmount a fly from Bellymount is very useful. However, one crucial point is to be able to mount flies on bellymount in the first place. Please add a video (and illustrations?) on how to install the system, and install the fly. Along these lines, it is unclear how flies can be fed on bellymount.

# Author comment: Author response to Decision Letter 1

**DOI:** 10.1371/journal.pbio.3000567.r003

8 Jan 2020

Attachment Submitted filename: KoyamaLAJ_Rebuttal.docx Click here for additional data file.

# Editor report: Decision Letter 2

**DOI:** 10.1371/journal.pbio.3000567.r004

**Authors:** Ines Alvarez-Garcia

13 Jan 2020

Dear Dr O'Brien,

On behalf of my colleagues and the Academic Editor, Bruce Edgar, I am pleased to inform you that we will be delighted to publish your Methods and Resources in PLOS Biology. 

The files will now enter our production system. You will receive a copyedited version of the manuscript, along with your figures for a final review. You will be given two business days to review and approve the copyedit. Then, within a week, you will receive a PDF proof of your typeset article. You will have two days to review the PDF and make any final corrections. If there is a chance that you'll be unavailable during the copy editing/proof review period, please provide us with contact details of one of the other authors whom you nominate to handle these stages on your behalf. This will ensure that any requested corrections reach the production department in time for publication.

Early Version

The version of your manuscript submitted at the copyedit stage will be posted online ahead of the final proof version, unless you have already opted out of the process. The date of the early version will be your article's publication date. The final article will be published to the same URL, and all versions of the paper will be accessible to readers.

PRESS 

We frequently collaborate with press offices. If your institution or institutions have a press office, please notify them about your upcoming paper at this point, to enable them to help maximise its impact. If the press office is planning to promote your findings, we would be grateful if they could coordinate with biologypress@plos.org. If you have not yet opted out of the early version process, we ask that you notify us immediately of any press plans so that we may do so on your behalf.

We also ask that you take this opportunity to read our Embargo Policy regarding the discussion, promotion and media coverage of work that is yet to be published by PLOS. As your manuscript is not yet published, it is bound by the conditions of our Embargo Policy. Please be aware that this policy is in place both to ensure that any press coverage of your article is fully substantiated and to provide a direct link between such coverage and the published work. For full details of our Embargo Policy, please visit http://www.plos.org/about/media-inquiries/embargo-policy/.

Thank you again for submitting your manuscript to PLOS Biology and for your support of Open Access publishing. Please do not hesitate to contact me if I can provide any assistance during the production process.

Kind regards,

Alice Musson

Publication Assistant, 

PLOS Biology

on behalf of

Ines Alvarez-Garcia,

Senior Editor

PLOS Biology
